# 2D Material‐Based Optical Biosensor: Status and Prospect

**DOI:** 10.1002/advs.202102924

**Published:** 2021-12-13

**Authors:** Zong‐Lin Lei, Bo Guo

**Affiliations:** ^1^ Key Lab of In‐Fiber Integrated Optics of Ministry of Education of China Harbin Engineering University Harbin 150001 China

**Keywords:** 2D materials, evanescent wave, fluorescence resonance energy transfer, optical biosensor, surface plasmon resonance

## Abstract

The combination of 2D materials and optical biosensors has become a hot research topic in recent years. Graphene, transition metal dichalcogenides, black phosphorus, MXenes, and other 2D materials (metal oxides and degenerate semiconductors) have unique optical properties and play a unique role in the detection of different biomolecules. Through the modification of 2D materials, optical biosensor has the advantages that traditional sensors (such as electrical sensing) do not have, and the sensitivity and detection limit are greatly improved. Here, optical biosensors based on different 2D materials are reviewed. First, various detection methods of biomolecules, including surface plasmon resonance (SPR), fluorescence resonance energy transfer (FRET), and evanescent wave and properties, preparation and integration strategies of 2D material, are introduced in detail. Second, various biosensors based on 2D materials are summarized. Furthermore, the applications of these optical biosensors in biological imaging, food safety, pollution prevention/control, and biological medicine are discussed. Finally, the future development of optical biosensors is prospected. It is believed that with their in‐depth research in the laboratory, optical biosensors will gradually become commercialized and improve people's quality of life in many aspects.

## Graphical abstract

The combination of 2D materials and optical sensors has become a hot research topic in recent years. Here, the recent progress of optical biosensors based on various 2D materials (graphene, black phosphorus, and MXenes) is reviewed. Furthermore, the applications of these sensors in biological imaging, food safety, pollution prevention and biomedicine are discussed. Finally, their future development trend is prospected.

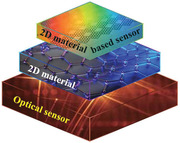

## 1 Introduction

Biosensor is a hot topic in today's scientific community. From electrochemical biosensor to optical biosensor, with the change of time and the development of science and technology, its sensitivity is higher and higher, and the detection of biomolecules is more and more extensive, which has made great progress in the medical, environmental, and even military fields.^[^ ^1^, ^2^ ^]^ The development of biosensors is a highly dynamic research field, which has developed rapidly in the past two decades. These different types of biosensors have been developed with breakthroughs in molecular biology, nanomaterials science and, most importantly, computer and optoelectronics.

**Figure 1:**
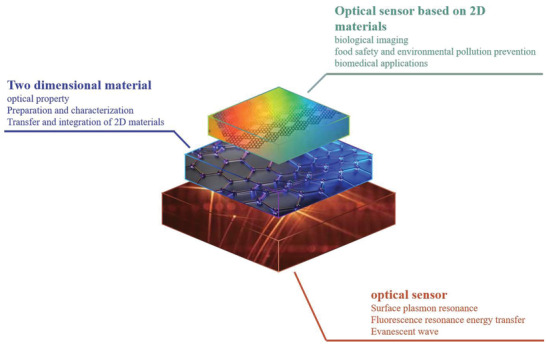
The basic framework of 2D material‐based optical biosensor.

In this period, optical biosensor has become a new field of biosensor, as shown in **Figure** **1**. For various biological molecules, such as DNA,^[^ ^3^ ^]^ RNA,^[^ ^4^ ^]^ virus,^[^ ^5^ ^]^ uric acid,^[^ ^6^ ^]^ protein,^[^ ^7^, ^8^, ^9^ ^]^ glucose,^[^ ^10^, ^11^ ^]^ dopamine,^[^ ^12^ ^]^ optical sensors have good adaptability. Meanwhile, 2D material is a new topic in recent years. With the in‐depth study of the optical properties of 2D materials,^[^ ^13^ ^]^ the optical sensors based on 2D materials have made great progress in recent years. In the field of food industry,^[^ ^14^ ^]^ environmental pollution monitoring,^[^ ^15^ ^]^ and medical treatment,^[^ ^16^ ^]^ optical sensors based on optical detection technology such as SPR, evanescent wave, and FRET have played an important role.^[^ ^17^, ^18^ ^]^ Using these methods, the combination of optical sensors and 2D materials shows the advantages of high sensitivity and high performance that traditional sensors do not have. As 2D materials have large specific surface area, good biocompatibility, and are suitable for high‐level surface interaction with biomacromolecules, the researchers have explored their application prospects in the field of biosensor, medicine, and drug delivery system.^[^ ^19^ ^]^

Here, we first introduce three common methods of optical sensors and the optical properties of 2D materials. Furthermore, various biosensors based on 2D materials are summarized in detail. Through the combination of 2D material and optical sensor, the performance of the sensor is greatly improved, which has a very broad prospect in the direction of biosensor. Finally, the applications and prospect of optical sensors based on 2D materials in biomedicine, food safety, and environmental pollution are discussed.

## 2 Fundamental of Optical Biosensor

Optical biosensor is a very popular research topic in recent years. It is a kind of sensor based on the transmission of light and collection of light signal. Compared with the traditional electrical sensor, it has the advantages of no electromagnetic interference, corrosion resistance, and high sensitivity. Here, we mainly introduce three main methods of optical biosensors: SPR, FRET, and microfiber‐based evanescent wave. In addition, the improvement of sensor sensitivity by 2D materials is also introduced.

### 2.1 Surface Plasmon Resonance

**Figure 2:**
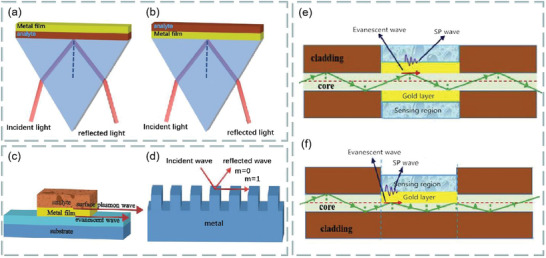
Different coupling structures of optical sensor. a) Otto configuration in prism coupling. b) Kretschmann configuration in prism coupling. c) Waveguide coupling. d) Grating coupling. e) Unclad/etched sensing structure in optical fiber coupling. f) Sensing structure in D‐shaped fiber coupling. Reproduced with permission.^[^ ^21^ ^]^ Copyright 2020, Elsevier.

Since the first SPR sensor was proposed by Liedberg et al. in 1983,^[^ ^20^ ^]^ it has become the most sensitive and label‐free technique for the detection of various molecular species in solution, which is of great significance in drug discovery, food safety, and biological reaction research.^[^ ^21^ ^]^ In principle, SPR is generated by surface plasmon excitation, which is produced by the interaction of free electron density oscillation and the electromagnetic wave between dielectric and metal film surface. When the evanescent wave and the surface plasma wave generated by light resonate with each other, the reflected light will be greatly weakened. According to the different excitation modes, it can be divided into four coupling modes: prism coupling, waveguide coupling, grating coupling, and fiber coupling, as shown in **Figure** **2**.

The prism coupling is shown in Figure 2a,b. Kretschmann structure lens is an upgrade of Otto structure lens. The metal film is located between the prism. The resonance conditions can be described as follows:

(1) $$k_{\mathrm{sp}}=\frac{w}{c}{\left(\frac{\varepsilon_{m}\varepsilon_{d}}{\varepsilon_{m}+\varepsilon_{d}}\right)}^{1/2}=k_{d}=\frac{w}{c}\sqrt{\varepsilon_{p}}\sin\theta$$

where *ε*_d_ and *ε*_m_ represent the dielectric constants of dielectric and metal films, respectively. *k*_sp_ is the propagation constant of surface plasma, *θ* is the incident angle, and *k*_d_ is the propagation constant of evanescent wave.

Prism is the most widely used optical coupling device in SPR research. It is composed of high refractive index nonabsorbable optical materials. Meanwhile, its bottom is coated with high reflectivity metal film, and under the film is dielectric. In recent years, it has been found that prism coupling has potential applications in many sensing fields.

Waveguide coupling is that when light is transmitted in a waveguide coupler, the propagation of light is based on total internal reflection, but part of the transmitted light exists in the form of evanescent wave. Under resonance condition, the evanescent wave passing through the metal film will be affected by the sample. Except that the structure is different from prism coupling, other principles are the same, as shown in Figure 2c.

The grating coupling (Figure 2d) can enhance the momentum of the incident light wave by inserting a diffraction grating into the sensing structure. When the incident light is incident on the metal dielectric interface at the angle of *θ* (resonance angle), the diffraction in the grating coupler can also increase the energy of the incident light. The coupling condition is as follows:

(2) $$k_{d}=k_{x}\pm \mathrm{mG}=\frac{2\pi }{\lambda }n_{d}\sin\theta \pm m\frac{2\pi }{\Lambda }$$

where *G* is the wave number of grating, *m* is the diffraction series, *k*_d_ is the constant value of diffraction light, Λ is the period of grating, *n*_d_ is the dielectric refractive index, grating is also a kind of optical coupling device widely used in SPR research.

Notably, prism‐, waveguide‐, and metal grating‐type have the characteristics of large volume, which limits their application in narrow and long‐distance measurement. On the contrary, the fiber coupling is compact and very suitable for sensing applications in narrow and long space. However, for the standard fiber, the evanescent field in the cladding is almost zero, so SPR cannot be excited. In order to generate SPR, it is necessary to ensure that part of the energy in the core can leak into the fiber cladding. Second, the thickness of the metal coating in the fiber should be moderate (≈30–50 nm). Finally, the polarization state of cladding mode should be controlled. At present, there are three kinds of fiber biosensors based on SPR: short‐period fiber grating biosensor, long‐period fiber grating biosensor, and fiber structure biosensor.^[^ ^22^ ^]^ The fiber structure can be divided into: hetero‐core sensing structure, unclad/etched sensing structure, D‐shaped sensing structure, tapered sensing structure, U‐shaped sensing structure, end‐face reflected sensing structure.

Figure 2e,f represents the uncovered/etched sensing structure and D‐type sensing structure in fiber coupling, and these two structures are also the most widely used structures. At present, there are many examples to use these two technologies to design multilayer high‐performance SPR fiber optic biosensors with high sensitivity, and can be numerically simulated by using the finite element method.^[^ ^23^ ^]^

Meanwhile, the methods to improve the sensitivity of SPR sensor have been developed rapidly. For example, because halloysite nanotubes improve the intensity of the detection field,^[^ ^24^ ^]^ the sensitivity of the measurement can be improved by modifying the SPR sensor with the characteristics of large surface area and high refractive index of halloysite nanotubes. In addition, the SPR sensor can also be optimized by the gold nanostructure. The performance of the optimized SPR biosensor is better than that of the traditional SPR biosensor.^[^ ^25^ ^]^ By transferring various 2D materials (such as graphene, transition metal dichalcogenides, black phosphorus, MXene) onto its surface, the sensitivity of SPR sensor can be greatly improved. In 2018, Wu et al. demonstrated that coating a small amount of Ti_3_C_2_T*_X_* Mxene on the surface of SPR biosensors could improve the sensitivity of the sensor.^[^ ^26^ ^]^ It is also found that MoS_2_ can be used as an effective adsorption layer for biological samples, but also plays an important role in the internal field. The combination of MoS_2_ and graphene can further improve the sensitivity.^[^ ^27^ ^]^ Therefore, plasma biosensor can be regarded as a powerful technology for quantitative determination of molecular analytes and biochemical reaction kinetics.

### 2.2 Fluorescence Resonance Energy Transfer

**Figure 3:**
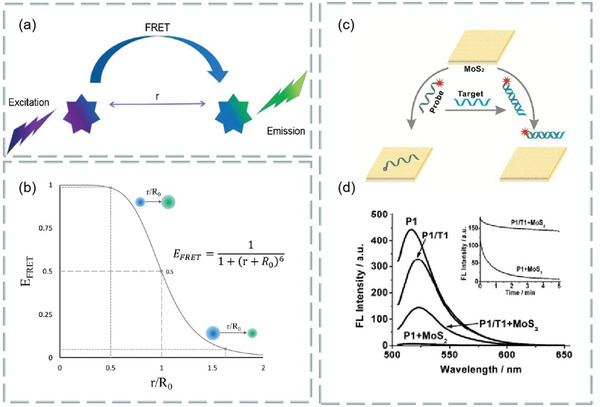
a) Diagram of fluorescence resonance energy transfer (FRET). b) The function diagram of energy transfer efficiency *E* and *r*/*R*_0_. Reproduced with permission.^[^ ^28^ ^]^ Copyright 2017, Elsevier. c,d) Selectivity of 2D materials as fluorescence quenching agents. Reproduced with permission.^[^ ^33^ ^]^ Copyright 2013, American Chemical Society.

FRET is a near‐field energy transfer from fluorescent donor to fluorescent receptor. In principle, under certain excitation, the emission of fluorescent donor can be absorbed by the nearby fluorescent receptor, resulting in fluorescence quenching phenomenon.^[^ ^28^ ^]^ The performance of FRET analysis mainly depends on three factors: fluorescent donor, fluorescent receptor, and the distance between donor and receptor. In order to generate fluorescence, the excitation band of the receptor must overlap with the emission band of the donor. Meanwhile, the distance between the excitation band and the emission band of the donor must be within the typical range on the nanoscale. Thus, the energy communication will occur in the near field, resulting in the energy transfer from the donor to the nearby receptor. As shown in **Figure** **3**a, the energy efficiency is as follows:

(3) $$E=\frac{1}{1+{\left(\frac{R}{R_{0}}\right)}^{6}}$$

where *R* is the distance between the donor and the receptor, and *R*_0_ is the distance between the donor and the receptor when the transfer efficiency is 50%. As shown in Figure 3b, it is defined as half of the energy transfer efficiency, which is determined by the following formula:

(4) $$R_{0}^{6}=\frac{9\left(\ln\;10\right)}{128\pi^{5}N_{A}}\frac{k^{2}Q_{D}}{n^{4}}J$$

where *n* is the refractive index of the medium, *Q*_D_ is the quantum yield of the donor without acceptor adsorption, *k* ^2^ is the dipole angle orientation factor of donor and acceptor molecules, *N_A_* is the Avogadro constant, and *J* is the spectral overlap integral of donor receptor pair.

For biosensors, it is very important to find a suitable fluorescence quencher. At present, the quenching agents can be divided into dynamic quenching agents and static quenching agents. For the former, the fluorescence intensity decreases due to the interaction between quencher and excited molecules. The latter refers to the process that the quencher forms a nonluminescent complex with the ground state fluorescent molecules to reduce the fluorescence intensity. The commonly used fluorescent quenching agents are metal ions, carbon nanotubes,^[^ ^29^ ^]^ and newly developed 2D materials.

The use of 2D materials as fluorescence quenching agents has become the focus of researchers. Among them, graphene and its derivatives have been proved to be very suitable for fluorescence quenching agents.^[^ ^30^ ^]^ Furthermore, other emerging graphene‐like 2D nanomaterials have been widely used as fluorescent groups or quenchers.^[^ ^31^ ^]^ The large surface area of single‐layer transition metal dichalcogenides not only enables biomolecules to carry out high physical adsorption on their surfaces, but also has the ability to quench the fluorescence of fluorescent clusters efficiently.^[^ ^32^ ^]^ The selective 2D fluorescence quencher can be more interesting to researchers. Study found that MoS_2_ can absorb the labeled dye single‐stranded DNA (ssDNA) probe.^[^ ^33^ ^]^ Due to the van der Waals force between the nucleobase and the basal plane of MoS_2_, the fluorescence of the dye is extinguished (Figure 3c). When the fluorescence probe was mixed with the prepared MoS_2_ nanosheets, the fluorescence of the FAM‐labeled ssDNA probe (P1) was almost completely extinguished. When P1 hybridized with the same amount of complementary target DNA T1 to form dsDNA, most of the fluorescence was still observed in the presence of MoS_2_, which indicated that the 2D material was selective as a fluorescence quenching agent (Figure 3d). In addition, WS_2_ has also been used as an efficient nanoquencher to develop novel fluorescent resonance energy transfer sensors.^[^ ^34^, ^35^ ^]^ Similarly, other 2D materials can also be used as fluorescence quenching agents, such as black phosphorus and 2D metal elements.^[^ ^36^ ^]^

In recent years, it has been found that 2D materials such as graphene, graphene oxide, WS_2,_ and MoS_2_ can be used as platforms for energy receptors or donors. Their ability to interact with the target is the key factor for the successful implementation of these sensors. Despite many positive aspects, their shortcomings, such as lack of unified size, poor quantum yield, poor fluorescence reproducibility, chemical stability and dispersion, need to be solved. Therefore, it may not be quantifiable in many cases. In addition, the applications of other 2D materials, such as borophene, silicene, phosphorene, and MXenes in the field of FRET, have also great potential and need to be further explored in the future.^[^ ^37^ ^]^

### 2.3 Evanescent Wave

Microfiber‐based evanescent wave biosensor is a kind of sensor based on the evanescent wave generated by the total reflection of light wave in the optical fiber. In principle, evanescent wave is an exponentially attenuated electromagnetic field. When light passes through the fiber core through total internal reflection, it produces a very small penetration depth at the interface, and interacts with the nearby medium, resulting in changes in the intensity, phase, or frequency of evanescent wave and light propagating in the fiber. By detecting these changes, we can obtain the information of biomolecules.^[^ ^38^, ^39^, ^40^, ^41^ ^]^ The field strength of evanescent wave may not change to zero, and it decays exponentially in the low index cladding:

(5) $$E=E_{0}\exp\left(-\frac{\delta }{D_{p}}\right)$$

where *δ* is the distance to the interface, *D*_p_ is the transmission depth, which can be expressed as:

(6) $$D_{p}=\frac{\lambda }{2\pi }\frac{1}{\sqrt{n_{1}^{2}\sin^{2}\theta -n_{2}^{2}}}$$

where *n*_1_ and *n*_2_ are the refractive indices of the media on both sides of the total reflection interface, and *λ* is the wavelength of the incident light. It can be seen that the transmission depth depends on the angle between the incident light and the normal of the interface.

**Figure 4:**
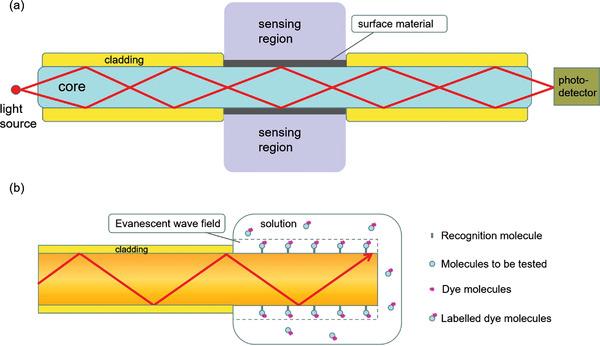
a) Schematic diagram of three‐layer evanescent wave‐based fiber optic sensor for hemoglobin detection using graphene layer. Reproduced with permission.^[^ ^38^ ^]^ Copyright 2018, Elsevier. b) Schematic diagram of evanescent wave fluorescence sensor.

When 2D materials are combined with evanescent wave sensors, the traditional two‐layer sensors (i.e., core and sample) will become new three‐layer sensors (core, sample, and surface material). Thus, the penetration depth (*D*_p_) will also be affected. We take graphene as an example, and a three‐layer fiber optic evanescent wave biosensor is shown in **Figure** **4**a.^[^ ^38^ ^]^ Then, the *D*_p_ of evanescent wave in the analytical medium is expressed as follows:

(7) $$D_{p}=\frac{\lambda }{2\pi \sqrt{n_{3}^{2}\sin^{2}\left[\sin^{-1}\left(\frac{n_{1}}{n_{3}}\sin\theta \right)\right]-n_{2}^{2}}}$$

where *λ* is the wavelength of light; *n*_1_, *n*_2,_ and *n*_3_ represent refractive index of core, analyte and surface material (i.e., graphene) respectively, and *θ* is the angle of incident light measured in normal direction from the core‐cladding interface.

The application of evanescent wave sensor can be extended to biological defense, disease diagnosis, biomedical and biochemical analysis.^[^ ^42^ ^]^ For example, in medical treatment, evanescent‐wave‐based optical biosensor has become an attractive choice for clinical nucleic acid screening. In this case, it adheres an almost unlimited range of biological identification probes to the sensor surface accurately and firmly by covalent surface chemistry method, so as to achieve accurate detection.^[^ ^43^ ^]^

Furthermore, the combination of evanescent wave and fluorescence is expected to be widely used in the field of optical biosensor.^[^ ^44^ ^]^ Evanescent wave is used to excite the fluorescent dye labeled on the surface of fiber core, so as to detect the properties and content of biomaterials attached to the surface of fiber core within the evanescent wave field range through specific reaction.^[^ ^45^ ^]^ As shown in Figure 4b, a section of optical fiber probe is used for biomolecule detection. In the sensing section, the fiber core and the sample solution to be measured form a total reflection interface, and the surface of the fiber core is attached with biometric molecules. When the biomolecule labeled with fluorescent dye reacts specifically with the biometric molecule, the fluorescent dye is fixed on the surface of the fiber core, enters the evanescent wave field and is excited to fluorescence. In this case, the content of biomolecules can be obtained by detecting the intensity of the excited fluorescence signal.

## 3 Properties, Preparation, and Integration Strategies of 2D Materials

2D materials, such as graphene, transition metal dichalcogenides, black phosphorus, MXenes, hexagonal boron nitride,^[^ ^46^ ^]^ and other 2D materials (metal elements, metal oxides, and degenerate semiconductors), are a newly developed class of nanomaterials with excellent properties in recent years, which have attracted huge attention in physics and material science. Next, we will introduce the properties, preparation, and integration strategies of 2D materials, so as to lay a foundation for their application in optical biosensors in the next part.

### 3.1 Properties of 2D Materials

**Figure 5:**
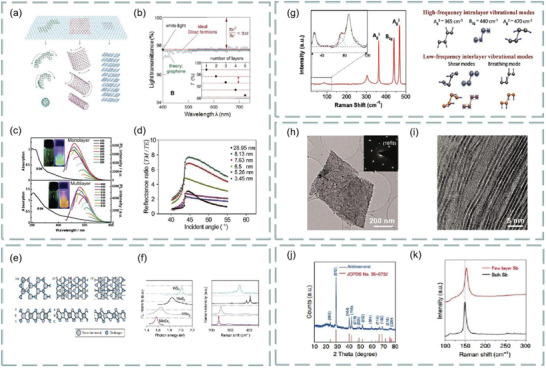
a) Graphene and its derivatives, including the 0D, 1D, and 2D allotropes of graphene. Reproduced with permission.^[^ ^47^ ^]^ Copyright 2012, Wiley‐VCH. b) Transmission spectrum of monolayer graphene. c) Photoluminescence of single‐layer and multilayer graphene films. Reproduced with permission.^[^ ^48^ ^]^ Copyright 2017, Wiley‐VCH. d) Angle‐dependent reflectance ratio plots of different thicknesses of reduced graphene oxide. Reproduced with permission.^[^ ^50^ ^]^ Copyright 2014, American Chemical Society. e) Coordination characterization of triangular prism (2H), octahedron (1T), or dimer (1T′). f) Photoluminescence and Raman spectra of four typical single‐layer semiconductor transition metal dichalcogenides. Reproduced with permission.^[^ ^53^ ^]^ Copyright 2013, Springer Nature. g) Typical Raman spectra and high‐ and low‐frequency internal vibration modes of black phosphorus. Reproduced with permission.^[^ ^58^ ^]^ Copyright 2017, Wiley‐VCH. h) TEM images of MXene nanosheets. i) Single‐layer and double‐layer MXene images. Reproduced with permission.^[^ ^59^ ^]^ Copyright 2011, Wiley‐VCH. j) Crystal structure of antimonene. k) Raman spectra of few‐layer Sb and bulk Sb. Reproduced with permission.^[^ ^60^ ^]^ Copyright 2017, Wiley‐VCH.

Graphene is a single atom thick carbon material, composed of carbon atoms bonded by SP^2^, arranged in 2D honeycomb strips. Generally, carbon has three basic forms: 0D, 1D, and 3D,^[^ ^47^ ^]^ as shown in **Figure** **5**a. Graphene can be further derived into graphene oxide and reduced graphene oxide. For optical applications, the transmission spectra of graphene are shown in Figure 5b, where the red line is the transmittance of 2D Dirac fermions.^[^ ^48^ ^]^ Clearly, it is a function of white light transmittance and the number of graphene layers. For graphene oxide, its intensity of luminescence varies with the number of layers. The photoluminescence of single‐layer and multilayer graphene oxide nanosheets is shown in Figure 5c. Meanwhile, the degree of oxidation affects the loading rate and photoluminescence quenching efficiency of single‐stranded oligonucleotides.^[^ ^49^ ^]^ Interestingly, the chemical properties of reduced graphene oxide are stable. Study found that the different thickness of reduced graphene oxide affects the reflectivity of light on the surface,^[^ ^50^ ^]^ and is related to the incident angle, as shown in Figure 5d.

Transition metal dichalcogenides are MX_2_‐type semiconductors, M represents transition metals, and X represents chalcogenide elements. They have unique optical and photoelectric properties.^[^ ^51^, ^52^ ^]^ Single‐ or multilayer transition metal dichalcogenides are direct bandgap semiconductors. Their bandgap energy and carrier type (n‐type or p‐type) vary with the composition, structure, and size of the compounds.^[^ ^53^ ^]^ Because of the different coordination spheres of transition metal atoms, transition metal dichalcogenide exists in many structural phases. Among them, the three common structural phases are characterized by the coordination of triangular prism (2H), octahedron (1T), or dimer (1Tʹ), as shown in Figure 5e. In addition, the photoluminescence and Raman spectra of four typical single‐layer semiconductor transition metal dichalcogenides are shown in Figure 5f.

Black phosphorus is a 2D monolayer with direct bandgap, which has excellent electro‐optic properties and high transporter versatility. Study found that the absorption of light induced by a few‐layer black phosphorus excitons decreases with increasing thickness, while the continuous absorption near the band edge is almost constant, independent of thickness. This condition has been proved to be similar to the quantum *σ*_0_ = *e* ^2^/4ℏ of the universal optical conductivity of graphene.^[^ ^54^ ^]^ Meanwhile, black phosphorus has a layer‐dependent bandgap which can be modulated from 0.3 eV (volume) to 2.0 eV (monolayer), so it has strong absorption in the ultraviolet and near‐infrared regions,^[^ ^55^ ^]^ and has significant in‐plane anisotropy.^[^ ^56^ ^]^ Importantly, black phosphorus is a kind of infrared layered material, which has a great future in the field of infrared photonics and photoelectron sensing.^[^ ^57^ ^]^ Typical Raman spectra of black phosphorus are shown in Figure 5g, including high‐ and low‐frequency component and their vibrational motions.^[^ ^58^ ^]^

MXene is a new type of 2D material, which was prepared by Gogotsi team of Drexel University in 2011 by stripping Ti_3_AlC_2_.^[^ ^59^ ^]^ Due to its good hydrophilicity and biocompatibility, MXene has been used as an ideal material to improve the sensitivity of biosensors. The general formula of MAX phase can be expressed as M_*n*+ 1_AX_*n*_, where M is the transition metal, A is IIIA or IVA group element, X is C and/or N, in the octahedral position, the M‐layer is almost surrounded by X atoms, and the atom layer is sandwiched between the M_*n*+ 1_X_*n*_ layers. In the M_*n*+ 1_AX_*n*_ phase, the metal bond between M_*n*+ 1_X_*n*_ layers is much stronger than the van der Waals interaction in traditional layered compounds such as graphene and transition metal dichalcogenides. Clearly, TEM images of exfoliated MXene nanosheets are shown in Figure 5h. The illustrations show SAD patterns, confirming the hexagonal symmetry of the plane. Single‐ and double‐layer MXene are shown in Figure 5i.

2D metal elements have attracted huge attention in recent years. Among them, antimonene is a new 2D material. Study found that its bandgap varies with the thickness of the layer, especially for the single‐layer antimonene, the theoretical prediction of the bandgap is 2.28 eV. Compared with graphene, antimonene has higher carrier mobility. The crystal structure of antimonene can be determined by XRD spectrum. As shown in Figure 5j, the diffraction peak of antimonene is the same as that of *β*‐Sb precursor. In addition, the Raman spectra of typical few‐layer Sb and bulk Sb (Figure 5k) can be measured to further study the crystal structure and quality of antimonene. Clearly, it has been shown that high quality single‐layer antimonene can be prepared by epitaxy.^[^ ^60^ ^]^

2D metal oxides have attracted great attention in various biological applications because of their unique physical and chemical properties, such as high photothermal response, temperature superconductivity, photoluminescence, flexibility, unique catalytic ability, and relatively low plasma regulation ability.^[^ ^61^ ^]^ For example, bulk TiO_2_ is a simple inorganic compound with four basic crystal types and is a wide bandgap semiconductor with a bandgap of ≈3 ev. Interestingly, its 2D nanosheets increased by 3.65 eV. The structural change from 3D to 2D leads to thickness limitation, resulting in a “blue shift” in the UV–Vis absorption spectrum of 2D nanosheets. Thus, TiO_2_ nanosheets have high surface area, easily adsorbed dye molecules, and promote electron transport.^[^ ^62^ ^]^

2D degenerate doped semiconductors are a new kind of plasma materials. Among them, the most representative research is H*_x_* MoO_3_ in plasma, such as ultrahigh capacity of H^+^ based on MoO_3_ nanodisk. Study found that free electrons similar to the Drude model produce plasmon resonance in the whole visible range and part of the near‐infrared region in H*_x_* MoO_3_, which means that the plasma resonance wavelength of H*_x_* MoO_3_ can be transferred to the visible and near‐infrared region, which has a very large spectral range and can be adjusted by intercalation H^+^ concentration.^[^ ^63^ ^]^

Graphene, transition metal dichalcogenides, black phosphorus, and MXene, these 2D semiconductors have far field forbidden band less than 2.0 eV, which greatly limits their applications, especially in the blue and ultraviolet light range of photoelectric devices. Led by antimonene, bismuthene, and arsenene^[^ ^64^ ^]^ represent a new undeveloped area of single element monolayer, and also have broad prospects and great challenges in optical biosensing. Based on the first principle calculation, arsenene and antimonene have wide bandgap and high stability, and these materials change from indirect bandgap semiconductors to direct bandgap semiconductors under small biaxial strain. This change in electronic structure paves the way for optoelectronic devices working in blue or ultraviolet light and sensors based on novel 2D crystals.^[^ ^65^ ^]^ As an optical biosensing platform, 2D honeycomb materials (phosphorene, arsenene, antimonene, and bismuthene) as sensitive platform can be used to optimize optical biosensors with high sensitivity and selectivity,^[^ ^66^ ^]^ indicating that these 2D metal elements have great potential in the field of optical sensing.

### 3.2 Preparation of 2D Materials

**Figure 6:**
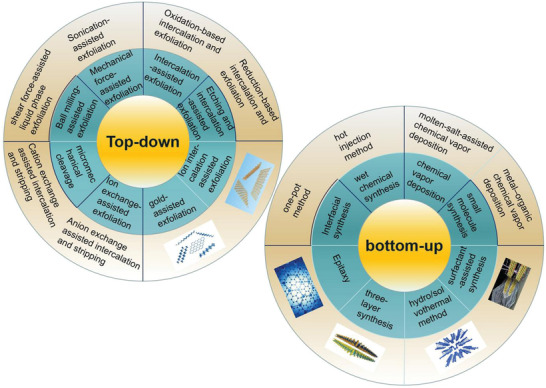
Top‐down and bottom‐up approaches of 2D materials.

As a rapidly developing hot field, the preparation of 2D materials is an important problem. At present, the top‐down method of exfoliating lamellar crystals into layered nanosheets has been widely studied.^[^ ^67^, ^68^ ^]^ In addition, other attractive bottom‐up methods, including chemical vapor deposition and wet chemical synthesis, have also attracted great attention.^[^ ^69^, ^70^ ^]^ Most of the top‐down methods rely on the stripping of thin‐layer 2D crystal from its parent layered body, so it can be widely used. In contrast, chemical vapor deposition growth and wet chemical synthesis are based on the chemical reactions of some precursors under specific experimental conditions.^[^ ^21^ ^]^ In this section, the popular 2D material preparing methods are summarized in recent years, as shown in **Figure** **6**. The main idea from top to bottom is to peel the layered material into 2D material, and then assemble the film on the substrate. On the contrary, the main idea from bottom to top is to use chemical reaction to rearrange the atoms between substances to prepare nanoparticles for film formation. **Table** **1** clearly summarizes the advantages, disadvantages, and prospects of the widely used methods for preparing 2D materials.^[^ ^19^, ^71^, ^72^, ^73^, ^74^, ^75^ ^]^

**Table 1:** The preparing technology of 2D materials

Method	Classification	2D materials	Advantage	Shortcoming	Prospect
Micromechanical cleavage		Graphene, black phosphorus,^[^ ^76^ ^]^ h‐BN, transition metal dichalcogenide,^[^ ^53^ ^]^ antimonytin,^[^ ^64^ ^]^ topological insulator,^[^ ^77^ ^]^ h‐MO^[^ ^78^ ^]^	Wide range of applications, no chemical substances and chemical reactions, clean surface	Production efficiency is low, the production speed is slow, and the size, thickness and shape are difficult to control	Improved micromechanical cleavage are needed
Mechanical force‐assisted exfoliation	Sonication‐assisted exfoliation	Graphene, transition metal dichalcogenide, h‐BN,^[^ ^79^ ^]^ 2D metal oxide, antimonene, black phosphorus,^[^ ^77^ ^]^ topological insulator	Simple and can be used to produce ultrathin 2D nanomaterials in solution at low cost	Yield of single‐layer nanosheets is low, and the transverse size of nanosheets is relatively small	
	Shear force‐assisted liquid phase exfoliation	Graphene, black phosphorus, transition metal dichalcogenide, etc	Preparation speed is fast, and low cost	Not very mature and needs further research	Large scale commercial production
Ion intercalation‐assisted liquid exfoliation	Chemical reagents that can insert different ions	Transition metal dichalcogenides, graphene and most other 2D materials	High efficiency	Residual chemical reagent	Mass production
Ion exchange‐assisted intercalation and exfoliation	Cation exchange assisted intercalation and stripping	Layered metal oxide and layered metal phosphorus trihalogenation (MnPS_3_、CdPS_3_)	High yield and large scale	Stripping of certain types of layered compounds; Layered double hydroxides nanosheets are difficult to separate	High‐yield and large‐scale production
	Anion exchange assisted intercalation and stripping	Layered double hydroxides^[^ ^80^ ^]^			
Intercalation‐assisted expansion and exfoliation	Oxidation‐based intercalation and exfoliation	Graphene oxide, h‐BN, TiO_2_^[^ ^61^ ^]^	Graphene oxide film	Strong oxidants may not be safe	
	Reduction‐based intercalation and exfoliation	Transition metal dichalcogenides、graphene、reduced graphene oxide	High yield	Decompose at high temperature	
Etching and intercalation‐assisted exfoliation		MXenes^[^ ^81^, ^82^ ^]^	Large quantities	Relatively dangerous	Mass production
Chemical vapor deposition		H‐BN nanosheets, topological insulators, NiCo_2_O_4_,^[^ ^83^ ^]^, transition metal dichalcogenides^[^ ^84^ ^]^	High crystal quality and purity		Large number
Wet chemical synthesis		Graphene, metal coordination polymer, transition metal dichalcogenides, ZnO^[^ ^61^ ^]^	Simple, repeatable, controllable and widely used	Experimental conditions are strict	industrial application
Gold‐assisted exfoliation^[^ ^85^ ^]^		Graphene, black phosphorus, transition metal dichalcogenides and RuCl_3_	No pollution, one step in place, large area and high quality monolayers	Technical support are needed	Large‐area monolayer

In the preparation of these 2D materials with atomic thickness, folds are inevitably produced in order to make them stable. However, it is meaningful that the existence of fold structures sometimes has a positive effect on 2D materials. In particular, the structure of fold structures in 2D materials also leads to six‐element properties, which is important for their sensing applications.^[^ ^86^ ^]^ At present, the pace of 2D material research is greatly accelerated, but in the process of identifying the peeling thickness of 2D material in optical contrast until monolayer, the image features of similar contrast are prone to false positives, while manual distribution is prone to error. Standard optical microscopy with CCD cameras can solve this problem. Not only can it be used as an analytical tool, but also can accurately determine the coverage, residue, pollution concentration and number of layers of the presented 2D materials, opening a new way to achieve fast and reliable automatic distribution.^[^ ^87^ ^]^

### 3.3 Integration Strategies of 2D Materials

The integration of 2D materials and optical devices is a key problem for biosensors. So far, some of the methods used to interact with 2D materials can be classified into four categories: one is to grow nanoscale 2D materials on a substrate by chemical growth. The substrate is then removed by etching or physical method and transferred to the optical sensor.^[^ ^70^ ^]^

Specific transfer methods can be divided into wet transfer and dry transfer. Among them, the etching method with polymethyl methacrylate as the transfer medium is very important. It can realize the large‐scale transfer of 2D materials, but there are some significant problems. First, this method needs to etch the metal substrate, which costs too much and wastes a lot of resources, and it is not conducive to large‐scale industrial application. Second, the surface of the 2D material transferred by this method will be contaminated by etching liquid metal ions and polymethyl methacrylate residues may not be completely removed, which will seriously affect the properties of 2D materials and the performance of related devices. The other is to mix the dispersions of 2D materials with organic compounds, pour the solution onto the substrate, and then dry them into thin films and attach them to the surface of the sensor. Third, the 2D material is made into a dispersion liquid, which vertically sinks the optical sensor into the dispersion liquid. Meanwhile, the end of the fiber can be attached to the material. The last method uses in‐situ layer by layer deposition technology to deposit 2D materials on tilted fiber Bragg gratings.^[^ ^88^ ^]^

## 4 Optical Biosensors Based on 2D Materials

As a new type of optical sensor in the 21st century, optical biosensor has been greatly expanded because of its non‐destructive operation mode, high signal generation and reading speed, and the development of optical fiber technology in recent years. As more and more advanced technologies support the development of optical biosensors, their detection range has gone from macro to micro, especially various biomolecules, such as mercury ions, hydroxyl‐containing analytes,^[^ ^89^ ^]^ even pathogenic microorganisms,^[^ ^90^ ^]^ immunoglobulin G, glycoproteins,^[^ ^91^ ^]^ plasma cells.^[^ ^92^ ^]^ Thus, optical biosensors play an important role in the biological field due to their wide variety, ease of use and high sensitivity.

Traditionally, optical biosensors play a role in the detection of single biological macromolecules. Furthermore, if the structure of the optical sensor is modified, it can also accurately detect a variety of biological macromolecules. For example, the traditional heterogeneous‐core fiber has only one sensing channel, and improves it to a two‐channel structured fiber, which also maintains high sensitivity and accuracy in the detection of multiple sets of analytes.^[^ ^93^ ^]^ Because of its unique optical properties and biocompatibility, 2D materials have become a booster for the development of optical sensors.^[^ ^94^ ^]^ In recent years, the combination of optical sensors and 2D materials has become a major development trend. For example, a graphene‐based nanosensor not only supports two plasma modes in the terahertz band, but also can be used to detect cancer cells and glucose.^[^ ^95^ ^]^ This highly sensitive and tunable optical biosensor will bring a revolution to biosensor. Since the discovery of graphene by A. Geim and C. Novoshorov in 2004, 2D materials have been widely used in optical biosensors. Next, we will introduce various optical biosensors based on 2D materials in detail.

### 4.1 Optical Biosensors Based on Graphene

In recent years, graphene‐based optical biosensors have been developed rapidly. In the second section, we introduce SPR technology in detail and analyze the combination of graphene and SPR technology. Theoretically, in order to excite plasmon resonance in graphene, the material needs high doping level. For example, Rodrigo et al found that the infrared plasmon response of graphene multilayer stacking is similar to the highly doped monolayer response of graphene. Compared with the previously explored monolayer devices,^[^ ^96^ ^]^ the highly doped monolayer response of graphene retains mobility and supports plasmon resonance with higher oscillator strength. Study found that this approach can well excite the plasmon resonance in graphene.

**Figure 7:**
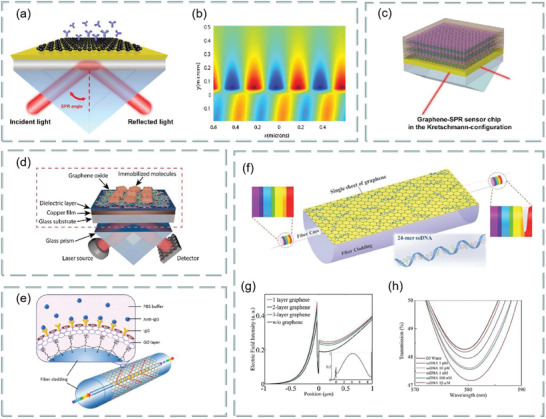
Optical biosensors based on graphene and its derivatives. a) Schematic diagram of the designed graphene‐bimetal SPR biosensor. The Au–Ag bimetal is formed by coating silver and gold films on a titanium film layer. b) Electric field distribution on the graphene‐bimetal sensing surface. Reproduced with permission.^[^ ^97^ ^]^ Copyright 2018, Elsevier. c) Kretschmann configuration of graphene oxide–Au substrate. Reproduced with permission.^[^ ^98^ ^]^ Copyright 2015, American Chemical Society. d) SPR biosensor chip based on copper dielectric plasma interface. Reproduced with permission.^[^ ^100^ ^]^ Copyright 2018, American Chemical Society. e) Schematic diagram of immune‐sensor for ultrasensitive unlabeled antibody‐antigen in dual‐peak long‐period grating. Reproduced with permission.^[^ ^101^ ^]^ Copyright 2017, Elsevier. f) Graphene gold hybrid plasma biosensor. ssDNA molecules are adsorbed on graphene monolayers by *π* stacking between aromatic rings and carbon atoms in honeycomb lattice. g) Variation of normalized electric field intensity of graphene with different layers. h) Change of transmission spectrum of graphene sensor in detecting ssDNA concentration. Reproduced with permission.^[^ ^102^ ^]^ Copyright 2017, Wiley‐VCH.

Both prism‐ and fiber‐coupling are the two most widely used methods in SPR technology. For example, based on prism coupling, Tong et al. proposed a novel graphene‐bimetallic plasma sensing platform for ultra‐high sensitivity phase interrogation in 2017,^[^ ^97^ ^]^ as shown in **Figure** **7**a. The hybrid structure combining graphene nanosheets and bimetallic films greatly enhances the sensitivity of the SPR sensor. Furthermore, the finite difference time domain method is used to study the electric field distribution on the graphene‐biometallic sensing surface, as shown in Figure 7b. In this case, even minor refractive index changes can lead to observable differential phase signals. To characterize the sensing performance of the designed graphene‐bimetallic substrate, phase sensitivity is defined as the ratio of differential phase change to refractive index change in the sensing medium as follows:

(8) $$S_{n}=\frac{\Delta \phi_{d}}{\Delta n_{s}}$$

where Δ*ϕ*_d_ is the difference phase between p and s polarized light, higher phase sensitivity corresponds to larger differential phase signals.

The graphene acts as an optical absorption medium, a large part of the excited light energy is transferred to provide resonance, more biomolecules are adsorbed on the sensing surface. In experiment, the peak phase sensitivity of 1.71 × 10^6^ deg per RIU was finally obtained in the wavelength of 632.8 nm. This sensing platform has great potential for detecting ultra‐low concentrations of small molecules or sample solutions.

In addition, based on prism coupling, Chung et al. also prepared graphene oxide and reduced graphene oxide films on gold films, their refractive index sensitivity was compared on SPR based sensors, and its aim is to reveal the role of graphene in plasma sensors. Finally, biomolecular sensing of graphene multilayers using BSA and anti‐BSA antibodies,^[^ ^98^ ^]^ as shown in Figure 7c. Interestingly, Verma et al. use Kretschmann structures to detect refractive index changes near the sensor surface, the sensitivity of the sensor is greatly improved compared with the traditional gold‐plated and sulfur prism sensors.^[^ ^99^ ^]^ Similarly, based on prism coupling, Stebunov et al. studied SPR biosensor chip based on copper dielectric plasma interface,^[^ ^100^ ^]^ as shown in Figure 7d. Study found that the thin copper film supports the excitation of surface plasmon and can be effectively coupled with external laser radiation. Thus, selective SPR biosensor analysis was realized by adsorbing biomolecules with graphene oxide on the surface.

Fiber coupling is another effective method. For example, Chen et al. investigated an ultra‐sensitive label‐free antibody–antigen immunosensor based on graphene oxide nanosheet functionalized biphasic long‐period grating.^[^ ^101^ ^]^ As shown in Figure 7e, it provides an excellent analytical platform for biological binding between prefixed IgG and target resistant IgG and can be used to detect the biological affinity of antibody to antigen in real time. Study found that, with the deposition of graphene oxide, the refractive index sensitivity of long‐period grating in low refractive index (1.333–1.347) and high refractive index (1.430–1.441) regions increased by 200% and 155%, respectively.

In addition, Li et al. use side‐polishing fibers with a gold film coated on the side surface, monolayer graphene was then transferred to the gold film to enhance the excited surface plasma and bind to biomolecules.^[^ ^102^ ^]^ Study found that graphene is especially helpful for the immobilization of ssDNA molecules. Driven by strong *π*‐stacking interactions between graphene aromatic carbon and bases, flexible ssDNA molecules try to maximize affinity and thus attach to graphene, so the whole molecule can fully interact with the surface plasma, resulting in an increase in the local refractive index. Thus, the phase‐matching point will be red‐shifted. In an example, the proposed biosensor is provided in 1 × 10^−12^–10 × 10^−6^ m Linear response over a wide detection range of logarithmic scale ssDNA concentration of 10^−6^ m, as shown in Figure 7f. Figure 7g shows the change in normalized electric field intensity of the surface plasmon excitations excited when graphene‐free layers, monolayer graphene, bilayer graphene, and triple‐layer graphene are deposited on 30 gold‐plated films. In addition, Figure 7h shows the change in transmission spectra when graphene‐enhanced plasmonic fiber sensors detect ssDNA concentrations.

In recent years, it has been found that localized surface plasmon resonance technology can also be used in optical biosensors. In principle, when the light is incident to the nanoparticles composed of noble metals, if the incident photon frequency matches the overall vibration frequency of the conduction electrons of noble metal nanoparticles or metal islands, the nanoparticles or metal islands will have a strong absorption effect on the photon energy, and local surface plasmon resonance will occur. For example, Chiu et al. proposed a modified gold nanoparticle/graphene oxide nanocomposite when graphene oxide sheets were modified with gold nanoparticles.^[^ ^103^ ^]^ In this case, localized surface plasmon resonance technology was introduced into the resonance energy transfer of spectral changes to detect two different interactions between proteins and hybrid nanocomposites.

**Figure 8:**
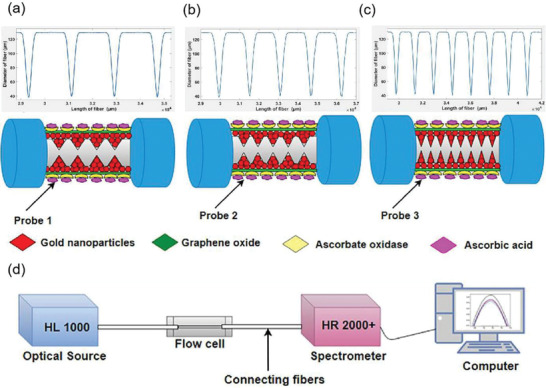
a) Four‐cone fiber probe. b) Five‐cone fiber probe. c) Eight‐cone fiber probe. d) Experimental setup. Reproduced with permission.^[^ ^104^ ^]^ Copyright 2020, Elsevier.

In addition, Zhu et al. demonstrated an ascorbic acid sensor based on the localized surface plasmon resonance technology,^[^ ^104^ ^]^ as shown in **Figure** **8**d, using a variety of tapered fibers (four, five, and eight cones) as probes (Figure 8a–c). The detection of ascorbic acid includes many aspects from preparing ascorbic acid samples with a concentration of 10 µm–1 mm to simulate the ascorbic acid levels present in the central nervous system. Then, a variety of test samples were prepared, i.e., 10 µm, 50 µm, 100 µm, 150 µm, 200 µm, 400 µm, 600 µm, 800 µm and 1 mm to study the performance of the sensing probe used to detect ascorbic acid, prepare the experimental setup as shown in Figure 8d. Using halogen tungsten white light source as light signal generator, record the localized surface plasmon resonance technology spectrum with a spectrometer. In an example, the proposed sensing probe was tested using the prepared test samples. Because of the ascorbic acid concentration, changes in the refractive index of the medium change the output wavelength, this phenomenon is called wavelength shift. According to this phenomenon, localized surface plasmon resonance technology biosensor based on graphene has ultrahigh sensitivity for ascorbic acid detection. Study found that the five tapered fiber has very good effect on detecting ascorbic acid and has superior performance. The sensing performance parameters, sensitivity, correlation correlator, and limit of detection are significantly enhanced, which are 8.3 nm mm^−1^, 0.9724 × 10^−6^ and 51.94 × 10^−6^ m, respectively.

**Figure 9:**
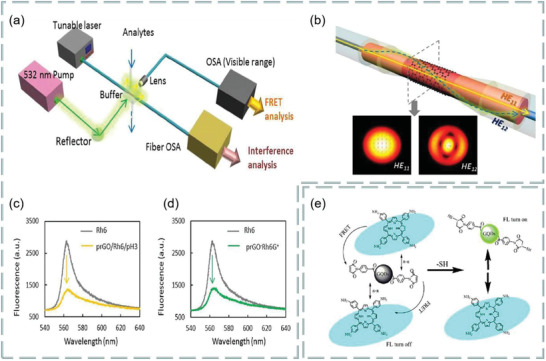
Graphene‐based FRET biosensor. a) Experimental setup. b) Structure of fiber mode‐interferometer. c) Fluorescence changes before and after dopamine detection. d) Fluorescence changes before and after detection of ssDNA. Reproduced with permission.^[^ ^105^ ^]^ Copyright 2016, Springer Nature. e) Chemical mechanism of biothiol detection based on FRET system. Reproduced with permission.^[^ ^108^ ^]^ Copyright 2020, Elsevier.

As mentioned in Section 2, FRET is a highly sensitive sensing detection method, which can be used to detect biomolecules such as dopamine, DNA, ascorbic acid, and biological mercaptan. Meanwhile, graphene oxide has high biological affinity because of its rich oxygen‐containing functional groups and chemical activity and is conducive to the binding of biomolecules and carboxyl groups. Thus, graphene oxide can be used as a fluorescence quencher and is very suitable for fluorescence resonance biosensing. For example, Yao et al. constructed an optical fiber FRET biochemical detection platform based on partially reduced graphene oxide by coating a partially reduced graphene oxide film on the surface of the optical fiber mode‐interferometer.^[^ ^105^ ^]^ In the experiment, rhodamine 6G was used as fluorescent donor, and partially reduced graphene oxide membranes was used as receptor, molecular adhesive, and evanescent field enhancer, which was coated around the optical fiber, so that Rh6G molecules were adsorbed on the surface of partially reduced graphene oxide. Because of partially reduced graphene oxide quenching, rhodamine 6G almost have no fluorescence scattering. Once a specific analyte is injected into the sampling area, with competition, rhodamine 6G molecules are released, and the recovery of fluorescence intensity can be observed. Meanwhile, in the process, effective refractive index of partially reduced graphene oxide coating varies with analyte concentration. Using optical mode‐interference, the phase difference can be accurately measured, as shown in **Figure** **9**a. The partially reduced graphene oxide coated fiber mode interferometer consists of a segment of multimode fiber sandwiched between two single‐mode fibers as shown in Figure 9b, the light interference between the HE_11_ and HE_12_ modes produces a resonant tilt of the spectrum. In addition, modification by different ions can be used to detect different biomolecules. The fluorescence before and after quenching is shown in Figure 9c,d. The detection limits were 1.3 × 10^−6^ m and 1 × 10^−12^ m, respectively. For DNA detection, Becheru et al. used a fluorescein‐labeled DNA probe to interact with a targeted single‐stranded complementary DNA on three‐layer graphene, to determine the most appropriate nucleic acid detection platform.^[^ ^106^ ^]^ This work provides a new idea for medical nucleic acid detection.

As we all know, vitamin C is an indispensable element in our body, a lack of vitamins C leads to many diseases. Thus, FRET use of vitamin testing is a hot topic. For example, Gao et al. developed a new open fluorescent sensor for the detection of ascorbic acid based on FRET between graphene quantum dots and square acid‐iron (III) squamate.^[^ ^107^ ^]^ Iron (III) can quickly cooperate with square acid to produce iron (III). The absorption band of square acid iron (III) overlaps greatly with the emission of graphene quantum dots, resulting in the fluorescence quenching induced by graphene quantum dots FRET. Therefore, the fluorescence sensing system can be applied to the direct analysis of ascorbic acid in practical samples. In the experiment, this FRET‐based nanosensor has an ascorbic acid concentration range of 1.0–95 × 10^−6^ m and showed high selectivity and high sensitivity response, and the detection limit was as low as 200 nm, which was lower than other fluorescence analysis methods.

Biothiols can be used as drugs, antidotes, and rubber vulcanizing promoters, and its sensitive detection is a great challenge in the biomedical field. It is found that the functional graphene quantum dots of maleimide can be synthesized according to the Michael addition principle, and the high‐efficiency Michael addition reaction between maleimide‐derived probe and biothio can be used for FRET identification of biothiols. For example, Jiangrong et al. established a FRET system with four, 4‐aminophenyl porphyrin for biothiol detection.^[^ ^108^ ^]^ In the presence of biothiols, the FRET process is closed because the fluorescence emission of graphene quantum dots follows the Michael addition mechanism. In principle, chemical mechanism of biothiol detection based on fluorescence resonance energy transfer system is shown in Figure 9e. The detection range is from 6.7 × 10^−10^ to 2 × 10^−7^, which limit is 2.34 × 10^−10^ mol L^−1^. Compared with a single detection system, FRET system has a wide detection range and low detection limit, and related biological molecules do not interfere with the quantitative identification of biological mercaptans. In this way, efficient determination of biothiols in food and human blood has become possible.

**Figure 10:**
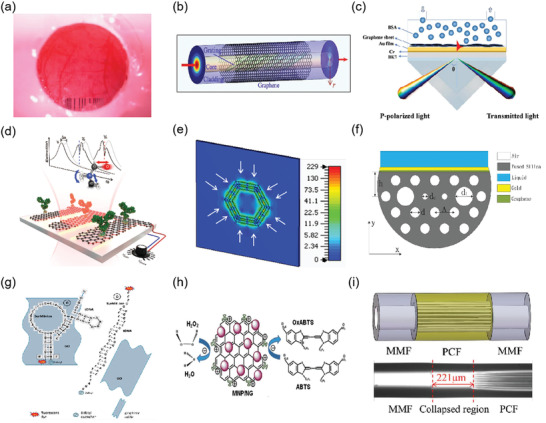
Various biosensors based on graphene and its derivatives. a) Bright‐field images of mouse cerebral cortex under cranial window. Reproduced with permission.^[^ ^109^ ^]^ Copyright 2014, Springer Nature. b) Tilted fiber grating based on graphene. Reproduced with permission.^[^ ^110^ ^]^ Copyright 2016, Optical Society of America. c) Graphene oxide sheets‐SPR sensor attached graphene. Reproduced with permission.^[^ ^111^ ^]^ Copyright 2018, American Chemical Society. d) Graphene‐based mid‐infrared plasma biosensor. Reproduced with permission.^[^ ^112^ ^]^ Copyright 2015, Science. e) Hexagonal optical antenna based on graphene. Reproduced with permission.^[^ ^113^ ^]^ Copyright 2018, Springer. f) D‐type photonic crystal fiber surface plasmon resonance biosensor composed of photonic crystal fiber, graphene, and metal. Reproduced with permission.^[^ ^114^ ^]^ Copyright 2018, Elsevier. g) The working principle of molecular beacon, the interaction between SurMB‐Joe and graphene oxide and tDNA. Reproduced with permission.^[^ ^116^ ^]^ Copyright 2018, MDPI. h) Graphical reaction of ABTS with H_2_O_2_ catalyzed by magnetic nanoparticle/nitrogen‐doped grapheme nanocomposites. Reproduced with permission.^[^ ^117^ ^]^ Copyright 2016, American Chemical Society. i) Multimode fiber‐photonic crystal fiber‐multimode fiber sensor structure. Reproduced with permission.^[^ ^118^ ^]^ Copyright 2018, Elsevier.

In addition to the above schemes, there are still many graphene‐based optical biosensor technologies. We know that the neural microelectrode array is transparent over a wide wavelength spectrum from UV to IR. Thus, the graphene‐based carbon layer electrode array device can be implanted into the brain surface of rodents for high‐resolution neurophysiological recording. **Figure** **10**a shows a bright‐field image under the cranial window of the mouse cerebral cortex under carbon layer electrode array micro‐ECoG device.^[^ ^109^ ^]^ In addition, using the polarization‐sensitive coupling effect between graphene and optical modes, the polarization modulation characteristics of graphene‐integrated over‐tilt fiber grating hybrid waveguides are also investigated,^[^ ^110^ ^]^ as shown in Figure 10b. Based on the traditional kreschmann structure, the schematic diagram of graphene oxide sheets SPR sensor attached with graphene^[^ ^111^ ^]^ is shown in Figure 10c. The sensitivity of SPR sensor is improved by spin coating graphene oxide sheets on the surface of gold film and related to the thickness of graphene oxide sheets coating, which can be customized by the spin coating time of graphene oxide sheets suspension. With the increase of spin coating times, the sensitivity first increased from 2257.9 to 2715.1 nm per RIU, and then decreased to 1194.7 nm per RIU. In the experiment, spin‐coated graphene oxide sheets can achieve a maximum sensitivity of 2715.1 nm per RIU three times, which is enhanced by 20.2% compared with uncoated shell. Due to the sensor with large surface area and rich surface functional groups, graphene oxide‐based SPR has higher sensitivity enhancement than volumetric solution in bovine serum albumin molecular detection (39.35%), which greatly improves the detection sensitivity of bovine serum albumin solution.

Infrared spectroscopy is the first choice for chemical identification using biomolecular vibrational fingerprints. For example, Rodrigo et al. proposed a highly sensitive and tunable plasma biosensor using the unique electro‐optic properties of graphene for chemical‐specific label‐free detection of monolayers with different mid‐infrared frequency proteins,^[^ ^112^ ^]^ as shown in Figure 10d. In addition, graphene‐based hexagonal optical antenna can also be used to enhanced biomolecular detection. Figure 10e depicts the relationship between pattern occurrence and the dielectric environment of hexagonal optical antenna.^[^ ^113^ ^]^ A dielectric structure size of 4000 nm produces bipolar modes due to the combination of quadrupole and octopole modes.

The highly sensitive SPR biosensor composed of D‐type photonic crystal fiber, graphene, and metal is shown in Figure 10f. Two small holes in the center of photonic crystal fiber facilitate the phase matching of core mode and plasma, while two large holes favor the birefringence and coupling of polarized light with metal. Specifically, the refractive index range is 1.34–1.40, the average sensitivity of the sensor is 4850 nm per RIU and the resolution is 2 × 10^−5^ RIU. Thus, this sensor has the advantages of long transmission distance, high sensitivity, and high resolution. For example, researchers use it for the real‐time detection of biomolecules and small drug molecules.^[^ ^114^ ^]^ Similarly, SPR sensors based on single‐mode fiber also have high sensitivity.^[^ ^115^ ^]^

The antiapoptotic protein survivin is highly expressed in human tumors and rare in normal adult tissues. It is one of the most promising tumor biomarkers. The supramolecular interaction between graphene oxide nanosheet nanocarrier and survivin molecular beacon realizes the functionalization of survivin molecular beacon by connecting fluorophore Joe and quencher Dabcyl (SurMB‐Joe). Figure 10g shows the principle of molecular beacon and the interaction of SurMB‐Joe with graphene oxide and tDNA.^[^ ^116^ ^]^

Colorimetry is another method for biosensors. Its principle is based on the color of the solution of the substance under test or the color of the colored solution formed after the addition of the chromogenic agent, and the content of the substance in the solution is determined according to the light intensity absorbed by the colored solution and the color depth in proportion to the substance content. Based on colorimetry, the researchers used the synthesized magnetic nanoparticles/nitrogen‐doped graphene nanocomposites to catalyze H_2_O_2_ and 2, colorimetric reaction of 2,2′‐azino‐bis (3‐ethylbenzo‐thiazoline‐6‐sulfonic acid) diammonium salt (ABTS). It was found that the typical absorption peaks associated with ABTS oxidation increased linearly with H_2_O_2_ concentration, up to 10 × 10^−3^ m, and a detection limit of 17.1 × 10^−6^ m. Since hydrogen peroxide is produced by glucose oxidase oxidizing glucose, this technique can detect the glucose concentration by detecting the concentration of hydrogen peroxide when the glucose addition reaches 18.0 × 10^−3^, the absorption intensity increased significantly, and the detection limit was 59.7 × 10^−6^ m, indicating the high performance of glucose detection.^[^ ^117^ ^]^ Magnetic nanoparticle/nitrogen‐doped graphene reaction between the catalytic ABTS and H_2_O_2_ of nanocomposites is shown in Figure 10h. Photonic crystal fibers with two‐stage multimode fibers were sputtered with gold film, then modified with graphene oxide and staphylococcus protein A for further immunosensor. In addition, the multimode fiber‐photonic crystal fiber‐multimode fiber sensor is shown in Figure 10i, where graphene oxide and SPA were coated on the photonic crystal fiber surface. Its refractive index sensitivity is 4649.8 nm/RIU, which is about 1888 nm per RIU higher than that without graphene oxide film and human refractive index, and the detection limit of IgG is as low as 10 ng mL^−1^.^[^ ^118^ ^]^

Leakage cavity mode resonance and lossy‐mode resonance are also very suitable for biosensors. For example, Wu et al. found that the leakage cavity mode resonance in periodic silicon nanowire arrays can be used as a biosensing platform with low cost, label‐free, and high sensitivity, and established a theoretical framework consistent with the experimental results for leakage cavity mode resonance phenomena.^[^ ^119^ ^]^ Study found that this sensor has a bulk refractive index sensitivity of 213 nm per RIU, and passes through the surface of graphene monolayer functionalized silicon nanostructure, which can be used to optically detect low concentration surface adsorption events.

The ability to maintain low‐level electronic noise is a major challenge for the development of graphene optoelectronic sensors, which is also the fundamental reason for limiting the resolution of image sensors. For example, operating graphene transistors in bipolar mode close to the neutral point can significantly reduce 1/*f* noise in graphene. Notably, this reduction of electronic noise is achieved through the simple sensing response of graphene chip, which greatly improves the signal‐to‐noise ratio compared with the transistor used to measure the conductor.^[^ ^120^ ^]^

### 4.2 Optical Biosensors Based on Transition Metal Dichalcogenides

**Figure 11:**
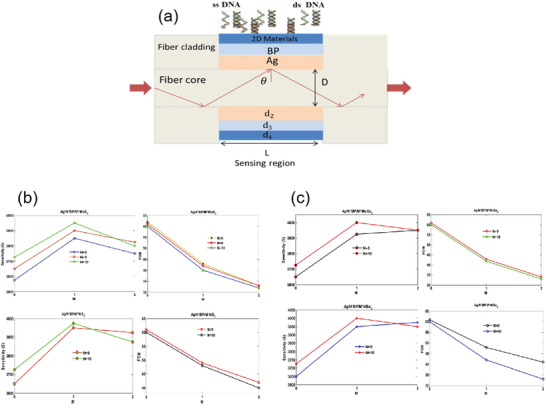
a) Schematic diagram of 2D biosensor based on 2D materials. b) Effect of MoS_2_ and WS_2_ layers on sensitivity and figure of merit of sensor. c) Effect of MoSe_2_ and WSe_2_ layers on sensitivity and figure of merit of sensor. Reproduced with permission.^[^ ^119^ ^]^ Copyright 2018, Elsevier.

Transition metal dichalcogenide structure was initially determined by Dickinson and Pauling in 1923.^[^ ^121^ ^]^ It has atomic‐scale thickness, direct bandgap, and good optoelectronic properties, which make its application in optical sensing become possible. For example, SPR based on fiber coupling, Rahman et al. theoretically designed a high‐performance fiber biosensor combining 2D materials such as graphene, MoS_2_, MoSe_2_, WS_2,_ and WSe_2_ with phosphorene.^[^ ^122^ ^]^ A biosensor based on various materials, as shown in **Figure** **11**a. In the theoretical model, the researchers chose a step refractive index multimode fiber as the cladding, the fiber consists of doped GeO_2_ silica core and pure silica. According to the Sellmeier dispersion relationship, refractive index of core and cladding depending on wavelength:

(9) $$n\left(\lambda \right)={\left(1+\frac{a_{1}\lambda^{2}}{\lambda^{2}-b_{1}^{2}}+\frac{a_{2}\lambda^{2}}{\lambda^{2}-b_{2}^{2}}+\frac{a_{3}\lambda^{2}}{\lambda^{2}-b_{3}^{2}}\right)}^{1/2}$$

where *a*_1_, *a*_2_, *a*_3_, *b*_1_, *b*_2_ and *b*_3_ is Sellmeier coefficients with values of 0.6961663, 0.4079426, 0.8974794, 0.0684043 × 10^−6^, 0.1162414 × 10^−6^, and 9.896161 × 10^−6^, respectively.

Figure 11b,c shows the effect of the number of layers of MoS_2_ (MoSe_2_) and WS_2_ (WSe_2_) on the sensitivity and figure of merit of the sensor. These transition metal dichalcogenides can improve the sensitivity and performance of optical biosensors. It is found that this optical fiber SPR biosensor can be used in medical diagnosis, enzyme detection, food safety detection and environmental monitoring.

#### 4.2.1 MoS_2_

**Figure 12:**
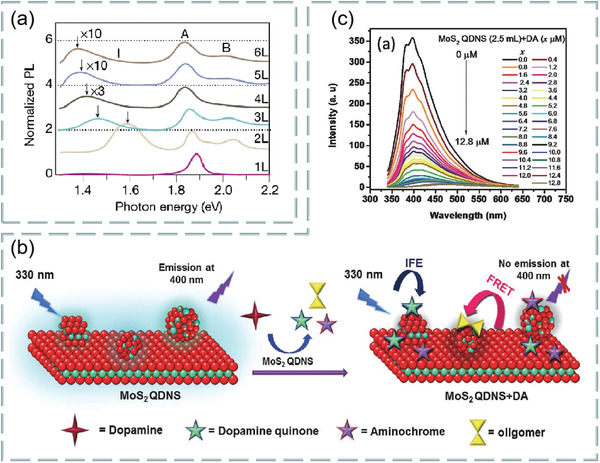
a) Normalized layer‐dependent photoluminescence spectra of MoS_2_. Reproduced with permission.^[^ ^123^ ^]^ Copyright 2019, Springer Nature. b) Schematic diagram of interaction between dopamine and MoS_2_ quantum dots dispersed over MoS_2_ nanosheets. c) Fluorescence response of MoS_2_ quantum dots with different concentrations of dopamine. Reproduced with permission.^[^ ^124^ ^]^ Copyright 2018, The Royal Society of Chemistry.

MoS_2_ is the most common transition metal dichalcogenides and their layer‐dependent photoluminescence spectra are shown in **Figure** **12**a. Among the three characteristic peaks, A and B are direct bandgap transitions and I are indirect transitions.^[^ ^123^ ^]^ From photoluminescence spectra, we can know that MoS_2_ is suitable for FRET technology. Comparing the fluorescence quenching efficiency of the two materials, it is found that the metal MoS2 nanosheet with 1t phase structure is higher. Meanwhile, metal MoS_2_ nanosheets have the ability to distinguish and adsorb single‐stranded DNA and double‐stranded DNA. Based on this, its application in fluorescent biosensor has broad development prospects. In addition, the application of MoS_2_ quantum dots in fluorescent biosensors is also a new idea.

For example, Mani et al. investigated a fluorescent sensor for selective and sensitive detection of dopamine in water samples by using FRET technology. In alkaline media, the MoS_2_ quantum dots dispersed over MoS_2_ nanosheets to form MoS_2_ nanohybrid materials as fluorescent material probes. The existence of dopamine makes the photoluminescence intensity of MoS_2_ quantum dots linearly quench with its concentration. At PH = 13, dopamine interaction with MoS_2_ quantum dots were dispersed on MoS_2_ nanosheets, as shown in Figure 12b. In addition, the fluorescence response of different concentration dopamine to MoS_2_ quantum dots is shown in Figure 12c. Study found that quantum dots dispersed over nanosheets sensor has high selectivity for dopamine, especially in the presence of ascorbic acid and uric acid, which is the most potential interference of dopamine in biological system. The sensitivity of this sensor is as low as 0.9 × 10^−9^ m, with two linear ranges from 2.5 × 10^−9^ to 5.0 × 10^−6^ m and 5.0 × 10^−6^ m to 10.4 × 10^−6^ m, which has significant ability in the analysis of real blood samples and shows good visual detection potential. The results show that the quantum dots dispersed on the nanochip sensor still show high selectivity for dopamine in the presence of ascorbic acid and uric acid. Thus, ascorbic acid and uric acid are most likely to interfere with the detection results of dopamine.^[^ ^124^ ^]^

**Figure 13:**
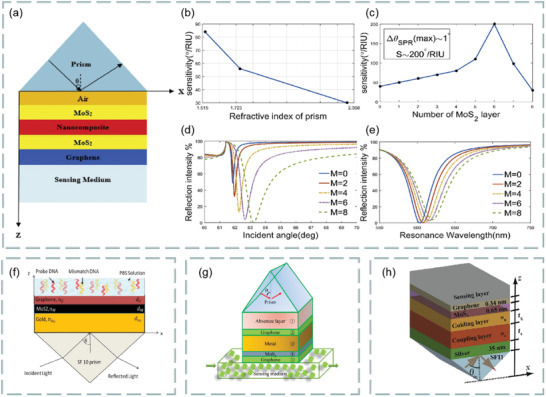
a) Structure diagram of optical biosensor based on graphene–MoS_2_. b) The change of sensitivity with the refractive index of prism. c) Sensitivity changes with MoS_2_ layers. d) The change of the light reflection intensity with respect to the incident angle at different number of MoS_2_ layers. e) SPR curve of spectral interrogation with different number of MoS_2_ layers. Reproduced with permission.^[^ ^126^ ^]^ Copyright 2019, Elsevier. f) Highly sensitive Au–MoS_2_–graphene hybrid SPR sensor. Reproduced with permission.^[^ ^127^ ^]^ Copyright 2017, Elsevier. g) Optical biosensor based on hybrid MgF_2_ prism and graphene–MoS_2_ layer. Reproduced with permission.^[^ ^128^ ^]^ Copyright 2018, Optical Society of America. h) Multilayer optical biosensor. Reproduced with permission.^[^ ^129^ ^]^ Copyright 2017, Elsevier.

With the deepening of MoS_2_ research, the synthesis of MoS_2_ nanocomposites has attracted great interest of researchers, and is expected to be applied in the fields of optical sensing, energy storage and conversion, and biomedicine.^[^ ^125^ ^]^ It is found that the hybrid structure based on graphene and MoS_2_ has important applications in the design and manufacture of high‐sensitivity biosensors. For example, Vahed et al. designed the air/MoS_2_/nanocomposite/MoS_2_/graphene heterostructure as a high‐sensitivity optical biosensor based on the SPR technology of Otto structure, as shown in **Figure** **13**a. Figure 13b shows the change of sensitivity with the refractive index of prism. Clearly, the higher the refractive index, the lower the sensitivity. Figure 13c shows the change of sensitivity with the number of MoS_2_ layers when the thickness of air layer is 35 nm the thickness of nanocomposite is 30 nm, and the number of graphene layers is 1–8, *f* = 0.85 (*f* is the filling factor, that is, the volume of metal nanoparticles, including the volume per unit volume of the host dielectric element). Figure 13d shows the variation of the light reflection intensity relative to the incident angle at different numbers of MoS_2_ layers (*M* = 0, 2, 4, 6, and 8). Figure 13e shows the SPR curve for spectral questioning of different numbers of MoS_2_ layers (M) at an air layer thickness of 30 nm and a graphene layer thickness of 35 nm.^[^ ^126^ ^]^ The best value sensitivity of biosensor takes six MoS_2_ layers and nanocomposite layers containing gold nanoparticles and TiO_2_ as the main dielectric, and the maximum sensitivity of 200° per RIU is realized. These results show that the hybrid structure of graphene–MoS_2_ plays an important role in improving the sensitivity of optical biosensors.

In addition, a highly sensitive Au–MoS_2_–graphene hybridized SPR biosensor for detection of DNA hybridization was also proposed,^[^ ^127^ ^]^ as shown in Figure 13f. By changing the SPR angle and the minimum reflection ratio, nucleotide bonding between double‐stranded DNA helical structures can be detected. Therefore, this sensor was able to successfully detect hybridization of the target DNA with probe DNA prefixed on the Au–MoS_2_–graphene hybrids, and has the ability to distinguish single‐base mismatch. In the experiment, it exhibits a high sensitivity of 87.8° per RIU, high detection accuracy of 1.28 and quality factor of 17.56. Furthermore, an experimental scheme using a low refractive index MgF_2_ prism to improve the sensitivity and performance of the sensor is also proposed,^[^ ^128^ ^]^ as shown in Figure 13g. Study found the introduction of low refractive index MgF_2_ prism and graphene–MoS_2_ hybrid layer, which greatly affects the field distribution in the sensing region, thus improving the performance of biosensors. In addition, a novel biosensor composed of a chalcogenide glass prism, Ag, coupling layer, guide layer, graphene–MoS_2_ hybrid structure, and analyte is proposed,^[^ ^129^ ^]^ as shown in Figure 13h. Compared with SPR sensing scheme, the sensitivity of the system is increased by 2 × 10^3^ times.

**Figure 14:**
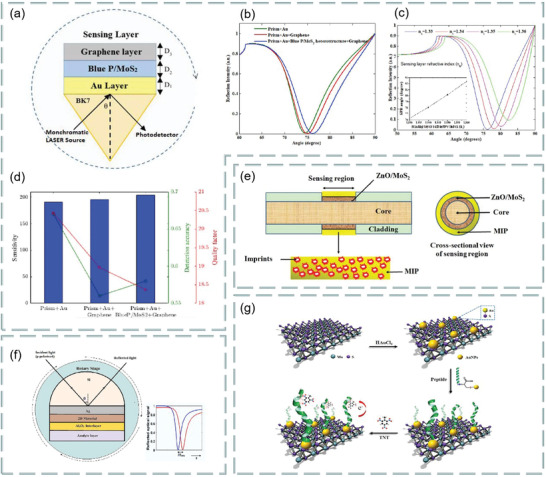
Optical biosensor based on MoS_2_ composite. a) Schematic diagram of a proposed SPR sensor configuration. b) Reflection curve of different materials. c) Reflection curves of simultaneous interpreting SPR sensors with different refractive index of sensing layer. d) A comparative study on sensitivity, detection accuracy and quality factor of different materials. Reproduced with permission.^[^ ^130^ ^]^ Copyright 2019, Elsevier. e) Optical fiber sensor based on loss mode resonance. Reproduced with permission.^[^ ^132^ ^]^ Copyright 2018, Elsevier. f) Optical sensor based on near infrared SPR. Reproduced with permission.^[^ ^134^ ^]^ Copyright 2018, Elsevier. g) The construction process of explosion detection biosensor based on nanocomposites.^[^ ^135^ ^]^ Copyright 2018, Elsevier.

Besides graphene–MoS_2_ heterostructure, the composites composed of MoS_2_ and phosphorene also have good application prospects in optical biosensors. It is found that the combination of MoS_2_ and blue phosphorene (P) into the heterostructure layer can improve the sensitivity of SPR sensor, even better than graphene SPR sensor. For example, Prajapati and Srivastava proposed a optical biosensing based on SPR technology—blue P/MoS_2_ heterostructure and graphene combination.^[^ ^130^ ^]^ As shown in **Figure** **14**a, p‐polarized light is incident on BK7 metal‐coated prism at a certain angle, and the incident angle is changed according to the angle inquiry method. However, the gold layer has poor absorption of biomolecules, which limits the sensitivity of conventional biosensors. To solve this problem, researchers directly coated the blue P/MoS_2_ heterojunction on the metal layer as the third layer, and coated graphene on the heterojunction layer. Thus, the graphene layer is in direct contact with the analyte and can be used to detect biomolecules. Figure 14b shows the reflection curves of the proposed SPR sensor (blue), graphene‐coated SPR sensor (red), and conventional SPR sensor (green) at 633 nm operating wavelength before biomolecules are adsorbed on the sensor surface. Figure 14c shows the reflection curve of the SPR sensor under the refractive index of different sensing layers. Figure 14d compares the sensitivity, detection accuracy, and quality factor of the proposed SPR sensor, graphene‐coated SPR sensor, and conventional SPR sensor. The results show that the proposed sensor has higher sensitivity (i.e., 204°/RIU) than conventional SPR sensor (i.e., 190.66° per RIU) and graphene‐based SPR sensor (i.e., 195.33° per RIU). Therefore, this work opens up a new way for the application of blue P/MoS_2_ heterostructure in SPR sensor.

Quasi‐2D MoS_2_ is a photoluminescent material with unique properties, which is easily affected by embedded ions. Therefore, it can be easily used to monitor biological systems, including the existence/exchange of important ions in organisms. Study found that it plays an important role in the intercalation of H^+^ and K^+^ ions. For example, Ou et al. selected glucose oxidase as a model to study ion transfer in the process of enzyme activity, and used quasi‐2D MoS_2_ nanoflakes to form a glucose‐sensitive biological system together with glucose oxidase.^[^ ^131^ ^]^ In principle, H^+^ ions and electrons produced by glucose oxidation are embedded into semiconductor MoS_2_ and converted into H*_x_* MoS_2_ (0 < *x* ≤ 1), so the photoluminescence characteristics of nanoflakes are manipulated.

Further experiments show that the existence of electric field promotes the ion embedding process, and the applied electrochemical force is the key factor for the operation of quasi‐2D MoS_2_ system. Interestingly, when the applied voltage is ‐0.5 and ‐1 V, about 5% of the small photoluminescence modulation in the system indicates that a small amount of H^+^ ions and electrons begin to diffuse and embed into the crystal structure of quasi‐2D MoS_2_; when the applied voltage is further reduced to – 1.5 V, the photoluminescence modulation increases significantly to ≈60%, resulting in almost complete photoluminescence quenching. This shows that a large number of generated H^+^ and electrons are embedded in quasi‐2D MoS_2_, which transforms it from semiconductor 2H phase to metal H*_x_* MoS_2_ phase, resulting in the loss of photoluminescence characteristics.

In recent years, there are many other MoS_2_‐based optical biosensors. For example, an optical fiber sensor based on loss mode resonance in the urine p‐cresol detection device is proposed,^[^ ^132^ ^]^ as shown in Figure 14e. In the experiment, the researchers immersed the ZnO/MoS_2_ nanosheet composite layer on the fiber core as the sensor layer and the molecularly imprinted polymer layer as the recognition medium. It should be pointed out that the sensor has the advantages of fast response, good stability, and good repeatability, and has a practical application prospect in the medical field. Interestingly, monolayer MoS_2_ and MoS_2_ crystals have no light absorption in the near‐infrared and mid‐infrared range and have very high dielectric constant, which makes them favorable for the study of lossless subwavelength photonics.^[^ ^133^ ^]^ Therefore, using the semiconductor–metal–dielectric heterojunction system, Sharma designed a high‐performance optical sensor based on near‐infrared SPR,^[^ ^134^ ^]^ as shown in Figure 14f.

In addition, experiments show that the sensor containing 2D composites such as graphene and MoS_2_ has greater figure of merit. Under irradiation, 2D MoS_2_ absorbs photons and generates broad‐spectrum carriers. Then, MoS_2_ is coupled with gold nanoparticles to generate enhanced electromagnetic field in the nanostructure, resulting in excellent optical properties. For example, using the biocompatibility of chemically modified nanoparticles with specific peptides, Wu et al. constructed an optical biosensor for explosion detection,^[^ ^135^ ^]^ as shown in Figure 14g. In short, as the most widely used member of the transition metal dichalcogenide family in optical biosensor, it is believed that the development of MoS_2_ will also drive the rapid development of other transition metal sulfides.

#### 4.2.2 WS_2_

**Figure 15:**
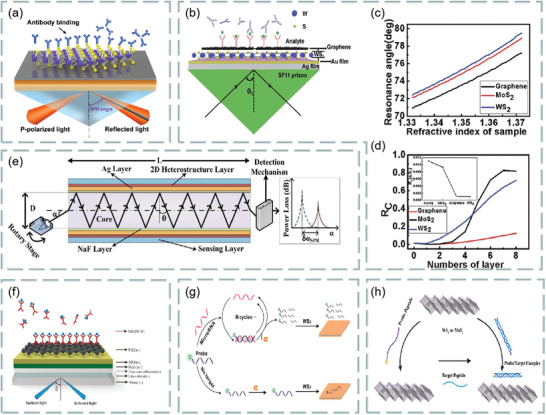
a) SPR biosensor based on transition metal dichalcogenide. Reproduced with permission.^[^ ^136^ ^]^ Copyright 2016, Springer Nature. b) Schematic diagram of the SPR sensor system. c) Resonance angle of the sensor based on bimetal film and graphene, MoS_2,_ or WS_2_ film relative to the refractive index of the analyte layer. d) Reflectivity varies with the number of layers of graphene, MoS_2,_ and WS_2_. The illustrations show the minimum reflectivity values for Au, Ag, graphene, MoS_2,_ and WS_2_. Reproduced with permission.^[^ ^138^ ^]^ Copyright 2017, Royal Society of Chemistry. e) Near‐infrared SPR sensor based on 2D heterostructure. Reproduced with permission.^[^ ^141^ ^]^ Copyright 2019, MDPI. f) SPR biosensor based on ITO–WS_2_ hybrid structure. Reproduced with permission.^[^ ^142^ ^]^ Copyright 2019, Springer. g) Detection of miRNA using WS_2_ nanoparticles as fluorescence quencher. Reproduced with permission.^[^ ^143^ ^]^ Copyright 2014, American Chemical Society. h) Optical biosensor based on WS_2_ and MoS_2_. Reproduced with permission.^[^ ^144^ ^]^ Copyright 2017, Springer Nature.

WS_2_ is a fine crystal or powder with metallic luster, belonging to hexagonal crystal system, semiconductor, and diamagnetic. In recent years, it has been found that few‐layer WS_2_ has unique advantages in optical biosensor. For example, Ouyang et al. designed a sensitivity enhanced surface plasmon resonance biosensor based on silicon nanosheet and transition metal dihalide by prism coupling,^[^ ^136^ ^]^ as shown in **Figure** **15**a. Its structure consists of six components: SF10 triangular prism, gold thin film, silicon nanosheet, 2D MoS_2_/MoSe_2_/WS_2_/WSe_2_ layers, biomolecular analyte layer, and sensing medium. The minimum reflectivity, sensitivity, and full width at half peak of SPR curve in visible and near‐infrared bands are studied theoretically by using Fresnel equation and transfer matrix method. The results show that WS_2_ silicon nanosheets have the highest sensitivity. In addition, Zhao et al. obtained similar results.^[^ ^137^ ^]^

Recently, Wang et al. developed a Kretschmann structure‐based biosensor composed of graphene, WS_2_ and Au–Ag bimetallic sheets by prism coupling, and further confirmed the excellent sensitivity performance of WS_2_,^[^ ^138^ ^]^ as shown in Figure 15b. Among them, SF11 prism is covered by silver layer, silver layer is covered by gold layer, and WS_2_ is located between gold layer and graphene layer, which together constitute SPR biosensor. To compare WS_2_ with MoS_2_ and graphene, the researchers used Fresnel equation and transfer matrix method to analyze the variation of reflectivity *R*_C_ at resonance angle (Figure 15c) and resonance angle with the number of layers of graphene, MoS_2_ and WS_2_, as well as the minimum *R*_C_ values of Au–Ag, graphene, MoS_2_, and WS_2_ (Figure 15d). The illustrations show the minimum reflectivity value of the bimetallic sensor based on WS_2_, which means that WS_2_ can more effectively promote light absorption. Therefore, the SPR sensor composed of WS_2_ and Au–Ag bimetallic film has the highest sensitivity among the three 2D materials. The results show that by depositing monolayer graphene and WS_2_ on Au–Ag hybrid nanostructures, the sensitivity is improved most, up to 182.5° per RIU, and the half‐height width is only 5.4°, which is better than the traditional sensor. Finally, it is found that the optimum thickness ranges of Ag layer and Au layer are 26–32, and 11–16 nm, respectively. Thus, the hybrid structure of graphene–WS_2_ and other 2D materials will have unexpected effects.

For glucose detection, Li et al. used g‐C_3_N_4_ and TiO_2_ nanosheets as new composites for constructing scaffolds for photoelectrochemical enzyme biosensors.^[^ ^139^ ^]^ In this way, the weak visible light excitation of TiO_2_ is improved and the photogenerated charge recombination on g‐C_3_N_4_ is delayed, so as to realize the enhanced response on the photoelectrochemical biosensor. In addition, Yang et al. also investigated polydopamine and graphene quantum dot modified TiO_2_ nanotubes to construct an ultrasensitive photoelectrochemical double electron acceptor biosensor.^[^ ^140^ ^]^ These methods have been successfully applied to develop photoelectrochemical glucose biosensor.

Besides the blue phosphorene–MoS_2_ heterostructure mentioned in the previous section, blue phosphorene–WS_2_ also has excellent results. These two kinds of near‐infrared monolayers with 2D heterostructures can be used as a good platform for the detection of biomolecules. It is found that the large merit values of the sensor design based on blue phosphorene/WS_2_ and blue phosphorene/MoS_2_ heterostructures are 7371.30 RIU^−1^ and 19179.69 RIU^−1^, respectively, and the optical properties of the former are better. In principle, the contact between the heterostructure and the analyte leads to an enhancement of the field at the interface and an increase in light absorption due to VDW attraction. For example, a fluorine‐based optical fiber sensor is suitable for the simulation and analysis of liver malignant tumors, and its structure is shown in Figure 15e.^[^ ^141^ ^]^

Other hybrid structures, such as SPR biosensor based on indium tin oxide (ITO)–WS_2_ hybrid structure, have significantly improved performance compared with traditional metal‐based SPR sensors,^[^ ^142^ ^]^ as shown in Figure 15f. This is because ITO is a mixture of In_2_O_3_ and SnO_2_, and has the advantages of adjustable dielectric constant, small capacitance, and high transmittance. Importantly, the transmittance of indium tin oxide film can reach 95% under visible light and 80% under infrared light. Thus, the sensitivity of functional materials of modulator and SPR can be improved. In the experiment, optimizing the thickness of Ag, ITO, and WS_2_ will further improve their sensitivity. The results show that the combination of ITO and WS_2_ will promote the development of biosensor.

The fluorescence resonance method based on WS_2_ nanosheets is also an important research field. Since WS_2_ nanosheets exhibit different affinity for short oligonucleotide fragments with single‐stranded duplex‐specific nuclease probes, so it is suitable as an effective quencher for adsorption fluorescence probe. Using this property, binding WS_2_ nanosheet fluorescence quenching and duplex‐specific nuclease signal amplification, Xi et al. proposed a simple, sensitive, and selective method for the detection of microRNA,^[^ ^143^ ^]^ as shown in Figure 15g. After adding the target microRNA, the single‐stranded DNA probe was hybridized with the target microRNA to form DNA/RNA heteroduplex. Because duplex‐specific nuclease only cuts single‐stranded DNA probes in DNA/RNA double strands, heterologous double strands will become substrates for duplex‐specific nuclease cleavage. In addition, the cleavage of single‐stranded DNA probe leads to the release of target microRNA and hybridization with another single‐stranded DNA probe, which will start the next round of cutting, release, and hybridization. Finally, this cyclic reaction produces a large number of fluorescence signals. In principle, oligonucleotides and the weak affinity on the nanosheets will not be adsorbed after binding with the weak affinity of the nanosheets. On the contrary, the single‐stranded DNA probe remained intact without duplex‐specific nuclease signal amplification reaction, and its fluorescence was almost completely extinguished due to its strong affinity with WS_2_ nanosheets. In the experiment, the detection limit of the sensor is as high as 300 × 10^−15^ m, and it can even distinguish the single base differences among the members of the microRNA family.

Biosensors based on MoS_2_ and WS_2_ are discussed separately, and their combination can be used as a new platform for fluorescent biosensors. For example, Sun et al. found that WS_2_ and MoS_2_ can significantly inhibit the fluorescence of arginine‐rich probe peptides, and the hybridization between probe peptides and their target collagen sequences leads to fluorescence recovery,^[^ ^144^ ^]^ as shown in Figure 15h. At the same time, the WS_2_‐based platform has high specificity for the target collagen peptide and is hardly disturbed by other proteins. Thus, using peptides as probe biomolecules can be used for the quantitative detection of collagen biomarkers in complex biological fluids. This is because the strong adsorption of WS_2_ or MoS_2_ on the fluorescent probe peptide quenched the fluorescence, and the hybridization between the probe and the target molecule restored the fluorescence. Therefore, this work provides an opportunity to build a new multifunctional biosensor platform.

#### 4.2.3 WSe_2_ and MoSe_2_

WSe_2_ and MoSe_2_ are also important members of transition metal dichalcogenide. However, compared with the above MoS_2_ and WS_2_, they are a lack of research in optical sensors. Interestingly, Wang et al. generated a transition metal disulfide hetero‐bilayer WSe_2_/MoSe_2_ with high carrier density by optical method and found the phase transition from interlayer exciton to charge‐separated electron/hole plasma by adjusting the optical excitation density above Mott threshold, in which the optically excited electrons and holes are localized to one single layer.^[^ ^145^ ^]^ This discovery not only opens the door to the optical control of electronic phase in 2D heterojunctions, but also lays a foundation for the development of these 2D materials in optoelectronic devices.

**Figure 16:**
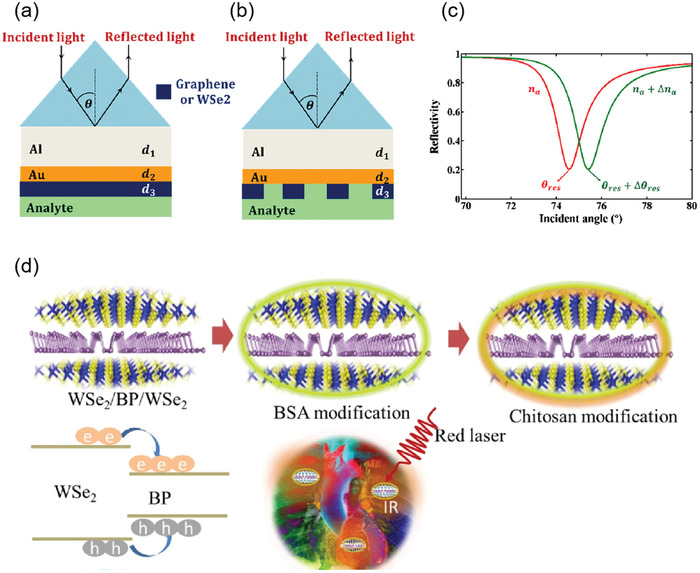
a) Graphene/WSe_2_ thin‐film optical sensor. b) Graphene /WSe_2_ nanobelt optical sensor. c) Schematic diagram of SPR reflection spectrum of optical sensor for two different analytes. Reproduced with permission.^[^ ^146^ ^]^ Copyright 2020, Springer. d) WSe_2_/black phosphorus/WSe_2_ single‐layer sandwich heterostructures are used for fluorescence imaging of biological species. Reproduced with permission.^[^ ^148^ ^]^ Copyright 2020, Springer.

As we know, the main structure of WSe_2_ consists of the upper and lower layers of selenium atoms connected to the middle layer of tungsten atoms. Moreover, WSe_2_ is thin and light, about 95% of the light passes through, but the remaining 5% of the light is absorbed by the material. Because of these unique optical properties, it also occupies a place in the field of optical biosensor. For example, Bijalwan et al. investigated a graphene‐WSe_2_ nanoribbon‐enhanced SPR sensor.^[^ ^146^ ^]^ Previous studies have shown that the sensitivity of continuous thin film sensor increases with the increase of the number of thin‐film layers. However, increasing the number of layers will also lead to the broadening of SPR spectrum and reduce the dip intensity. In the experiment, the maximum quality factor and detection accuracy were obtained by single graphene/WSe_2_ layer. Furthermore, the quality factor of graphene‐based sensor can be increased from 164.28 to 184.97 RIU^−1^, and 162.5 RIU^−1^ of WSe_2_‐based sensor to 181.11 RIU^−1^. Meanwhile, the detection accuracy of graphene was improved from 0.82 to 0.92, and WSe_2_ was improved from 0.81 to 0.90. **Figure** **16**a shows an optical sensor based on graphene/WSe_2_ film. Figure 16b shows an optical sensor based on graphene/WSe_2_ nanoribbon. Figure 16c shows a schematic diagram of the SPR reflectance spectra of optical sensor for two different analytes.

In addition, black phosphorus–WSe_2_ heterostructure has efficient light emission in the mid‐infrared band, and photoactivation and electroactivation provide a promising platform for the research and application of infrared light.^[^ ^147^ ^]^ Interestingly, the 2D vdws heterostructure follows the I‐band arrangement, allowing the transfer of electrons and holes from one complementary material to another. Thus, the 2D vdWs heterostructure is expected to enhance the radiative recombination of carriers, making it suitable for fluorescence imaging of biological tissues, cells, DNA, RNA, and protein. For example, Neupane et al. used WSe_2_/black phosphorus/WSe_2_ monolayer sandwich heterostructure to perform fluorescence imaging of biological species after treatment with bifluoromethane sulfonamide (TFSI),^[^ ^148^ ^]^ as shown in Figure 16d. In the experiment, bovine serum albumin was modified to solve the serious problem of how to excrete nanomaterials in vitro in medical applications. In this case, bovine serum albumin is a protein polymer, which further improves the surface compatibility of 2D nanomaterials, so that it can be excreted after use, and bovine serum albumin can also absorb toxic substances and heavy metal ions. Importantly, the WSe_2_/black phosphorus/WSe_2_ heterostructure emits strong near‐infrared emission and generates heat. Notably, in vivo imaging of tumor cells or tissues, it is necessary to protect healthy cells from thermal damage. Researchers found that the modification of 2D heterostructures by chitosan can solve the problem of thermal radiation of 2D vdws heterostructures. Therefore, in vivo biological imaging, bovine serum albumin, and chitosan modified WSe_2_/black phosphorus/WSe_2_ can achieve the best effect.

**Figure 17:**
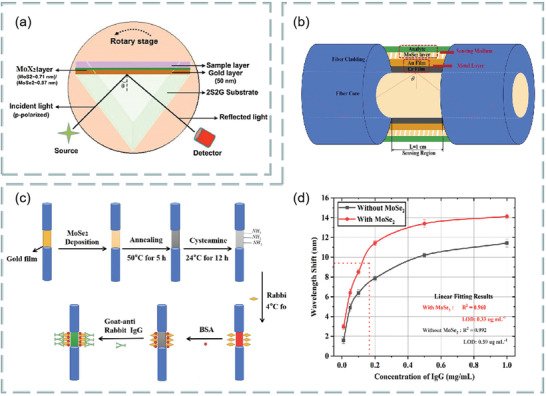
a) Near‐infrared plasma sensor with four‐layer structure. Reproduced with permission.^[^ ^149^ ^]^ Copyright 2018, Elsevier. b) SPR sensing structure for goat‐antirabbit IgG detection. c) Preparation and modification of MoSe_2_–Au membrane for IgG immunoassay. d) Response curves of goat antirabbit IgG with different concentrations. Reproduced with permission.^[^ ^150^ ^]^ Copyright 2019, Institute of Electrical and Electronics Engineers.

MoSe_2_ is another widely used 2D material, which can be used to detect specific reactions. For example, Sharma et al. proposed a near‐infrared plasma sensor based on chalcogenide as substrate, transition metal dichalcogenide and gold layer, in which MoS_2_ and MoSe_2_ monolayer structures are used,^[^ ^149^ ^]^ as shown in **Figure** **17**a. It is found that under the conditions of longer wavelength and single‐layer MoS_2_/MoSe_2_, the figure of merit is significantly improved, which provides a new idea for the future research of biochemical sensing. In addition, Liu et al. proposed an optical fiber SPR biosensor with MoSe_2_–Au nanostructure to detect goat anti‐rabbit IgG by combining the rapid response of optical fiber SPR sensor with the enhanced sensitivity of MoSe_2_ nanofilm,^[^ ^150^ ^]^ as shown in Figure 17b. The preparation and modification process of MoSe_2_–gold membrane for IgG immunoassay is shown in Figure 17c. Specifically, gold film is plated on the optical fiber, then MoSe_2_ is deposited to form cysteamine, and then rabbit IgG antigen is modified to block the non‐binding site, in which rabbit IgG and goat antirabbit IgG have binding effect. The response curve of goat to rabbit IgG of different concentrations (linear region in dotted line) is shown in Figure 17d. For this sensor, its sensitivity is 2821.81 nm per RIU, which is about 98.7% higher than the traditional SPR sensor without MoSe_2_. Furthermore, using bovine serum albumin as the target molecule, the biological affinity of the biosensor with MoSe_2_ deposition layer of 0–8 was tested. Weighing the sensitivity and the optimal value, the optimal deposition cycle of MoSe_2_ is four times, 2793.36 nm RIU^−1^, and 37.24 RIU^−1^, respectively. This rapid response and high biological affinity show that this MoSe_2_ Au SPR immunosensor has strong applicability in specific interaction and immunotherapy. 2D transition metal dichalcogenides have been widely used in biosensors, especially in the sensitive detection of nucleic acids, proteins, and biomolecules. Among them, SPR, fluorescence, and colorimetry based on these materials will become the focus of future research.^[^ ^32^ ^]^

In the above two sections, the research progress of graphene and transition metal dichalcogenides in optical biosensors is reviewed in detail. As the two most popular 2D materials, they have both similarities and different properties. For transition metal dichalcogenides, it is a kind of 2D material with indirect bandgap. Due to its unique structure, there is no dangling bond on its surface, the atoms in its layer are connected by covalent bond, and the layers are combined by van der Waals force.^[^ ^151^ ^]^ Thus, it is very suitable for assembling 2D material heterostructures, which is of great significance to improve the performance of optical devices. For graphene, it has no bandgap and low optical absorptivity (only 2.3%), so it cannot emit photoluminescence in the visible region and can only be used in low response sensors, for example, as an energy receptor for fluorescence quenching. Study found that the quenching distance of optical sensors based on graphene can reach 30 nm and the quenching efficiency can reach 100%, while the quenching distance of the traditional FRET system is about 10 nm.

### 4.3 Optical Biosensors Based on Black Phosphorus

**Figure 18:**
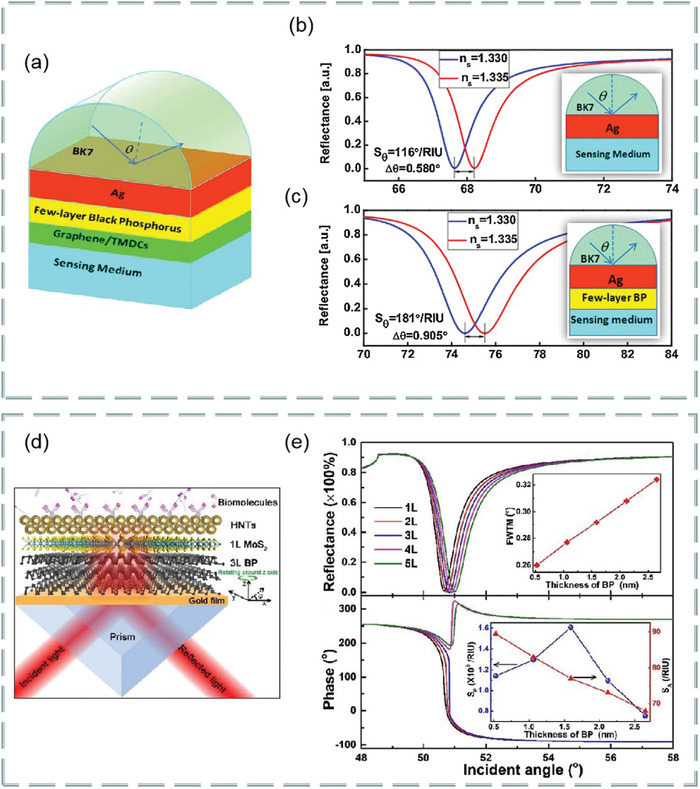
a) Sensitivity test of SPR sensor with black phosphorus/graphene/transition metal dichalcogenides hybrid structure. b) Structure diagram, intensity sensitivity, and phase sensitivity diagram of traditional sensors. c) Structure diagram, intensity sensitivity, and phase sensitivity diagram of sensor based on black phosphorus. Reproduced with permission.^[^ ^152^ ^]^ Copyright 2017, Elsevier. d) Biosensor based on halloysite nanotubes, MoS_2,_ and black phosphorus layers structure diagram. e) Variation of reflectivity and phase with incident angle and the number of black phosphorus layers. Illustration shows the SPR curve of black phosphorus film with different thickness, full width at tenth maximum change and detection sensitivity with angle and phase. Reproduced with permission.^[^ ^153^ ^]^ Copyright 2019, The Royal Society of Chemistry.

The combination of black phosphorus and SPR technology can improve the sensitivity and stability of the sensor, so it has a good application prospect. For example, using BK7 prism coupling, Wu et al. proposed a biosensor that can improve SPR sensitivity based on black phosphorus and graphene/transition metal dichalcogenides hybrid structure,^[^ ^152^ ^]^ as shown in **Figure** **18**a. In the experiment, the sensitivity of conventional SPR biosensor based on Ag is 116° per RIU (Figure 18b). Interestingly, the sensitivity of biosensor is greatly improved by using graphene, MoS_2_, WS_2_, MoSe_2_, WS_2,_ and other 2D materials as protective layers and coating them on the surface of black phosphorus. Due to the large refractive index of black phosphorus, the performance of the sensor coated with nearly 9 layers of black phosphorus is improved the most. For example, when black phosphorus with a thickness of 5 nm is coated on the traditional structure, the sensitivity can be as high as 181°per RIU. In addition, through prism coupling, Jia et al. proposed a highly anisotropic and ultrasensitive plasma biosensor by vertically stacking halloysite nanotubes, MoS_2_ and black phosphorus layer on the gold film,^[^ ^153^ ^]^ as shown in Figure 18d. When the number of layers of BP film is changed, the reflectivity and phase change with the incident angle, as shown in Figure 18e. Among them, the number of layers of MoS_2_ is 1, the refractive index of sensing medium is 1.333 RIU^−1^, and the thickness of halloysite nanotube and gold film are 400 and 40 nm, respectively. The illustration shows the variation of the full width of SPR curve and the sensitivity of angle and phase detection for black phosphorus films with different thicknesses, where ∆*n* = 0.002 RIU. Clearly, when the number of black phosphorus layers is 3, the angle detection sensitivity and phase detection sensitivity of the sensor reaches 77.0548 RIU^−1^ and 1.60595 × 10^5^ RIU^−1^, respectively. These results show that black phosphorus has a good application prospect in SPR biosensor.

**Figure 19:**
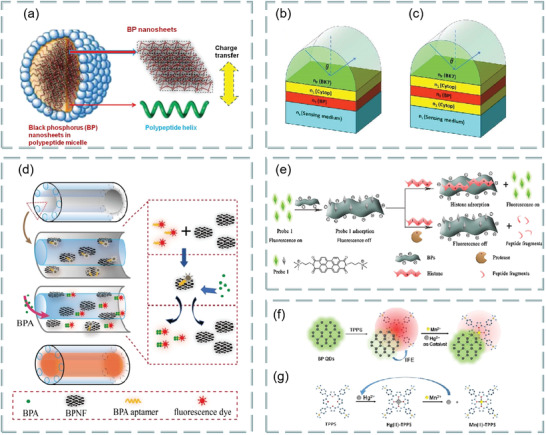
a) Black phosphorus nanosheets coated with peptide micelles. Reproduced with permission.^[^ ^154^ ^]^ Copyright 2019, American Chemical Society. b) Lossy‐mode resonance sensor without protective layer N_3_ (CYTOP). c) Lossy‐mode resonance sensor with CYTOP. Reproduced with permission.^[^ ^124^ ^]^ Copyright 2018, American Chemical Society. d) High selectivity detection and sensing platform for BPA based on fluorescence. Reproduced with permission.^[^ ^156^ ^]^ Copyright 2020, Elsevier. e) Schematic diagram of protease detection and analysis system. Reproduced with permission.^[^ ^157^ ^]^ Copyright 2019, Elsevier. f) Schematic diagram of IFE fluorescence colorimetric detection of Hg^2+^ based on black phosphorus quantum dots and TPPS. g) Catalytic effect of Hg^2+^ on the coordination reaction between Mn^2+^ and TPPS. Reproduced with permission.^[^ ^158^ ^]^ Copyright 2017, American Chemical Society.

In recent years, many biosensors based on black phosphorus have been developed. For example, because the optical properties of black phosphorus nanosheets are very suitable for biosensors, Kumar studied optically active polypeptide micelles, which can simultaneously passivate and encapsulate black phosphorus nanoparticles, integrate with biomedical equipment and apply to the fields of optical sensors and drugs,^[^ ^154^ ^]^ as shown in **Figure** **19**a. In particular, the optical properties of black phosphorus nanoparticles were not damaged in the preparation process, so they can be further used in phototherapy. For example, potential applications will include slightly adjusting the optical properties after cancer imaging to make it close to the visible range. It is believed that photodynamic therapy with black phosphorus will have great prospects for the treatment of cancer.^[^ ^155^ ^]^

Lossy‐mode resonance is a new technology. In principle, the traditional SPR sensor can only excite SPR with TM polarized light, while the TE polarized light and TM polarized light can realize lossy mode resonance. Thus, the high‐performance lossy mode resonance sensor based on few‐layer BP also has broad application prospects in the field of biosensors.^[^ ^124^ ^]^ As shown in Figure 19b,c, CYTOP is an amorphous fluoropolymer with low refractive index, which is widely used in SPR sensors. As a matching layer, it is located between the prism and BP film, which can enhance the electric field and effectively prevent the oxidation of black phosphorus. Compared with the traditional SPR sensor, the *Q* factor of black phosphorus‐based lossy‐mode resonance sensor with TE and TM polarized light is greatly improved. Especially for TM polarization mode, the highest *Q* factor of up to 2 × 10^5^ RIU^−1^ can be obtained, and has opened up a new direction in biosensing field. In addition, Qiao et al. used hollow antiresonance fiber technology and the physical properties of black phosphorus to ultrasensitively detect bisphenol A (BPA) in blood and environmental samples,^[^ ^156^ ^]^ as shown in Figure 19d. Theoretically, the hollow antiresonant fiber can not only realize broadband optical transmission but also restrict the light to the low refractive index liquid core, ensuring the maximum overlap between the light and the liquid core. Furthermore, the fluorescent‐labeled BPA specific aptamer is used to modify the inner surface of hollow antiresonance fiber to provide an intelligent sensing interface, and the high selective detection of BPA is realized by measuring fluorescence.

Proteases are closely related to many physiological processes and diseases. To this end, Hu et al. proposed a sensitive fluorescence detection and inhibitor screening method based on black phosphorus nanosheets,^[^ ^157^ ^]^ as shown in Figure 19e. Study found that the aqueous solution of perylene probe (probe 1) showed strong fluorescence. Interestingly, black phosphorus can absorb probe 1 through electrostatic interaction, resulting in fluorescence quenching of probe 1. Importantly, histone can control the interaction between perylene probe and black phosphorus, which can be further regulated by the introduction of protease. Thus, the activity of protease can be monitored by detecting the fluorescence intensity of probe and the principle of protease detection and analysis system. Note that this method does not need labeling, has high sensitivity and good selectivity.

In addition, in order to detect mercury ions, Gu et al. proposed a ratio measurement fluorescence sensor based on the internal filtration effect of tetraphenylporphyrin tetrasulfonic acid (TPPS) on black phosphorus quantum dots, in which high fluorescence black phosphorus quantum dots were synthesized from top to bottom by ultrasonic‐assisted sol‐gel thermal method,^[^ ^158^ ^]^ as shown in Figure 19f,g. In the presence of Hg^2+^, the inner filter effect (IFE) induced by the overlap of black phosphorus quantum dots and the TPPS absorption spectrum is suppressed, and the fluorescence of black phosphorus quantum dots is restored. Meanwhile, the red fluorescence of TPPS is quenched due to the coordination between TPPS and Mn^2+^. This sensor has a good linear response to Hg^2+^, ranging from 1 to 60 × 10^−9^ m, and the detection limit is 0.39 × 10^−9^ m. Thus, this method is suitable for the detection of Hg^2+^ in real samples.

Although the application of black phosphorus in optical biosensors is promising, it still has great challenges, especially its environmental stability is one of the major problems hindering its practical application.^[^ ^159^ ^]^ Recently, researchers have studied the degradation mechanism of black phosphorus by fast scanning micron Raman spectroscopy. It is found that there are two competitive processes in the reaction between oxygen and layered black phosphorus: edge degradation and surface degradation.^[^ ^160^ ^]^ Like other 2D materials, the application of black phosphorus in biosensors will become a research hotspot in the future. It can be expected that due to its unique optical properties, black phosphorus will become an ideal candidate for biomedical applications, biological macromolecular detection, photothermal therapy, drug detection, and biological imaging.

### 4.4 Optical Biosensors Based on MXenes

**Figure 20:**
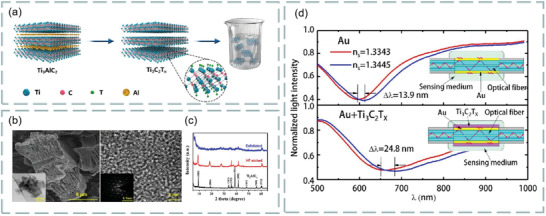
a) Preparation of Ti_3_C_2_T*_x_* MXene nanosheets by HF selective etching. b) SEM and HRTEM images of Ti_3_C_2_T*_x_* MXene delamination after HF corrosion, respectively. c) X‐ray diffraction patterns of initial Ti_3_AlC_2_ (black line), HF etched Ti_3_AlC_2_ (red line), and exfoliated Ti_3_C_2_T*_x_* (blue line). d) Variation diagram of normalized light intensity relative to wavelength of optical fiber sensor with MXene layer attached. Reproduced with permission.^[^ ^162^ ^]^ Copyright 2020, American Chemical Society.

Although MXene has been discovered for less than ten years, it has been widely used in the fields of biosensors, gas sensors, and humidity sensors because of its good biocompatibility, large specific surface area, and broadband light absorption,^[^ ^161^ ^]^ For example, Ti_3_C_2_T*_x_*‐MXene nanosheets prepared by selective etching of HF is shown in **Figure** **20**a. The SEM and HRTEM images of Ti_3_C_2_T*_x_*‐MXene delamination after HF corrosion are shown in Figure 20b. The illustrations are TEM and selective electron diffraction of Ti_3_C_2_T*_x_*‐MXene nanosheets, respectively. Figure 20c shows the X‐ray diffraction patterns of initial Ti_3_AlC_2_ (black line), HF etching Ti_3_AlC_2_ (red line), and spalling Ti_3_C_2_T*_x_* (blue line). Interestingly, when 2D material is attached to the metal film based on SPR sensor, its normalized light intensity will change. For example, Figure 20d shows the normalized change of light intensity relative to wavelength of an optical fiber sensor attached to MXene layer.^[^ ^162^ ^]^ When the measured refractive index changes from 1.3343 to 1.3445, the resonance displacement of the sensor without MXene layer is 13.9 nm and that of the sensor with MXene layer is Δ*λ* = 24.8 nm, which indicates that MXene layer can improve the sensitivity of optical fiber SPR sensor. MXene nanosheets functionalize graphene oxide via Ti–O–C covalent bonding to obtain MXene‐reduced graphene oxide sheets,^[^ ^163^ ^]^ which have great potential in the fields of portable electronic devices, flexible energy storage systems, and biosensors.

**Figure 21:**
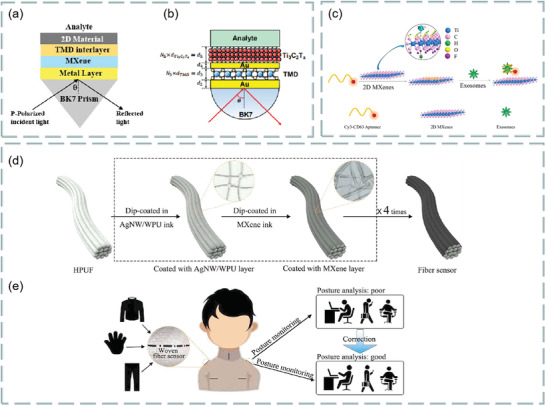
a) Multilayer SPR biosensor. Reproduced with permission.^[^ ^164^ ^]^ Copyright 2020, Elsevier. b) Schematic illustration of the SPR sensor with Ti_3_C_2_T*_x_* and 2D transition metal dichalcogenides. Reproduced with permission.^[^ ^165^ ^]^ Copyright 2019, MDPI. c) Fluorescence quenching and recovery of Ti_3_C_2_T*_x_*. Reproduced with permission.^[^ ^166^ ^]^ Copyright 2018, American Chemical Society. d) Schematic diagram of optical fiber strain sensor. e) Schematic diagram of human posture monitoring, analysis and correction system based on intelligent wearable fabric. Reproduced with permission.^[^ ^167^ ^]^ Copyright 2019, The Royal Society of Chemistry.

In recent years, MXene‐based sensors for detecting various biological macromolecules have developed rapidly. For example, Srivastava et al. proposed a SPR biosensor consisting of BK7 prism, MXene, transition metal dichalcogenides, black phosphorus, and gold (Au),^[^ ^164^ ^]^ as shown in **Figure** **21**a. In the experiment, the highest sensitivity of 190.22° per RIU was obtained using single‐layer nanomaterials. This work shows that MXene based sensor has great potential as a new biosensor. Similarly, based on BK7 prism, Xu et al. theoretically proposed a new SPR sensor based on Au‐Ti_3_C_2_T*_x_*‐Au transition metal dichalcogenides,^[^ ^165^ ^]^ as shown in Figure 21b. For aqueous solution, the refractive index sensitivities of the proposed SPR sensors of single‐layer Ti_3_C_2_T*_x_* MXene and four‐layer MoS_2_, five‐layer MoS_2_, five‐layer WS_2,_ and six‐layer WSe_2_ are 174° per RIU, 176° per RIU, 198° per RIU and 192° per RIU, respectively. Compared with the traditional gold film SPR sensor, the sensitivity of the biosensor using MXene and transition metal dichalcogenides integrated SPR is greatly improved.

In addition, Zhang et al. constructed a unique Cy3 labeled CD63 aptamer (Cy3‐CD63 aptamer)/Ti_3_C_2_ MXene nanocomposite based on FRET technology as a nanoprobe for quantitative detection of self‐standard ratio FRET of exosomes,^[^ ^166^ ^]^ as shown in Figure 21c. In the experiment, Cy3‐CD63 aptamer was mixed with MXenes aqueous solution and added in vitro. Furthermore, after adding Cy3‐CD63 aptamer to MXenes, its fluorescence was quenched and quickly recovered by adding exons.

With the gradual development of MXene, a new type of optical fiber biosensor‐flexible sensor has entered the eye of people. Meanwhile, the development of wearable medical electronic technology has greatly improved its sensitivity and scalability. Based on the stretched polyurethane fiber, it is easy to weave into wearable devices in conventional fabrics. For example, a multilayer sensing structure optical fiber sensor based on silver nanowire (AgNW)/waterborne polyurethane (WPU) layer and MXene layer is self‐assembled,^[^ ^167^ ^]^ as shown in Figure 21d. In principle, intelligent fabrics are produced by integrating optical fiber strain sensors into different clothes, so as to provide a prototype system for human posture monitoring, analysis, and correction for medical and health care applications. Figure 21e is a schematic diagram of human posture monitoring, analysis, and correction system based on smart wearable fabric. The system uses optical fiber sensors to stitch directly to different clothing materials. In the experiment, the sensor has both ultra‐high sensitivity (GF = 1.6 × 10^7^) and wide operating range (up to 100%), as well as high reliability and stability (1000 cycles), fast response (344 ms), and relaxation (344 ms).

### 4.5 Optical Biosensors Based on 2D Metal Elements and Oxides

**Figure 22:**
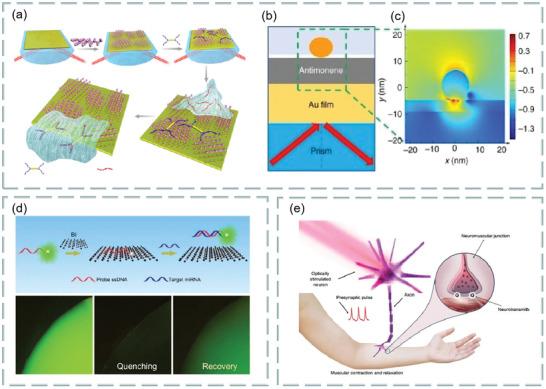
a) Fabrication of a microRNA sensor integrated with antimonene nanosheets. b) Electromagnetic field intensity distribution of individual AuNRs. c) Enhancement of AuNRs local electric field distribution. Reproduced with permission.^[^ ^169^ ^]^ Copyright 2019, Springer Nature. d) Schematic diagram and fluorescence change diagram of microRNA detection based on bismuthene nanosheets. Reproduced with permission.^[^ ^171^ ^]^ Copyright 2020, The Royal Society of Chemistry. e) Scheme of optical gene engineering neuron system and artificial photoelectric sensor motion device. Reproduced with permission.^[^ ^173^ ^]^ Copyright 2019, Springer Nature.

Recently, antimonene has attracted great interest as a single element 2D material. For example, Zhang et al. obtained high‐quality antimonene and antimonene quantum dots by electrochemical stripping and ultrasonic chemical stripping and studied the nonlinear optical response of antimonene in the visible light range.^[^ ^168^ ^]^ Further studies have shown that antimonene has strong stability compared with unstable black phosphorus. Especially in the field of biosensors, it also has important research value. For example, Xue et al. developed a SPR sensor based on antimonene, which was used to detect clinical biomarkers such as microRNA‐21 and microRNA‐155.^[^ ^169^ ^]^ Study found that more nonlocalized 5s/5p orbitals in antimonene greatly enhance the interaction with single‐stranded DNA, and single‐stranded DNA and microRNA complement each other to form double strands. The specific method is to first assemble antimonene nanosheets on the surface of the gold film, then adsorb on the antimonene nanosheets with AuNR‐single‐stranded DNAs, and then flow through the surface of antimonene with different concentration of microRNA solution, forming a double chain with complementary AuNR‐single‐stranded DNA. Furthermore, the interaction between microRNA and AuNR single‐stranded DNA resulted in the release of AuNR‐single‐stranded DNA in antimonene nanosheets. As can be seen from **Figure** **22**a, on the SPR surface, the decrease of AuNR‐single‐stranded DNA molecules significantly reduces the SPR angle. In addition, the electromagnetic field intensity distribution of a single AuNRs on 5 nm thick interlayer gold film is calculated by finite difference time domain method, as shown in Figure 22b. Figure 22c shows the enhancement of the local electric field distribution at AuNRs at the 632.8 nm plane when the incident wave plane is polarized along the X direction. It is worth noting that the detection limit of the sensor can reach 10 × 10^−18^ m, which is 2.3–10000 times that of the existing microRNA sensor. Therefore, it can be predicted that antimonene will promote the development of optical biosensors in the future.^[^ ^170^ ^]^

Bismuthene is a single element 2D material with high carrier mobility and room temperature stability. Recently, researchers have studied the fluorescence quenching mechanism of bismuth, that is, the weak fluorescence charge transfer in the ground state, by using femtosecond pump probe spectroscopy. It is found that bismuthene is a good fluorescence quenching material and is expected to play an important role in the field of biosensors. For example, the ultrathin sensing platform based on bismuthene can be used for the specific detection of microRNA and even identify single‐base mismatch, which provides a new idea for the detection of sensitive microRNA molecules in early cancer treatment,^[^ ^171^ ^]^ as shown in Figure 22d. Clearly, the fluorescence image of FAM‐single‐stranded DNA probe solution (10^−6^ m) mixed with bismuth is the brightest. When the FAM‐single‐stranded DNA probe solution is mixed with bismuth, the fluorescence image will be much darker due to quenching effect. After the FAM‐single‐stranded DNA was mixed with bismuth and miRNA‐21, the fluorescence was relit with the quenching agent Bi. The results show that the sensor can even recognize single‐base mismatch and the detection limit is up to 60 × 10^−12^ m.

TiO_2_ is nontoxic and has good opacity and brightness, so it can be used as a new sensing material.^[^ ^172^ ^]^ Besides the bulk form, TiO_2_ nanomaterials as 2D metal oxides have also been widely studied and widely used in the fields of energy and environment. In terms of biosensing, Akbari et al. proposed a new artificial biosensor photoelectric sensing motion system for controllable excitation of optogenetic engineering neurons in biological motion system,^[^ ^173^ ^]^ as shown in Figure 22e. The device is based on inorganic optical synapses (doped with TiO_2_ nanomaterials) assembled into liquid metal actuators. In principle, photoelectric synapses produce polarized excitatory and inhibitory postsynaptic potentials, trigger the vibration of liquid metal droplets and simulate the expansion and contraction of biological fibers, which can be applied to artificial nerve sensory movement, light‐driven neurorobot, microfluidic chip, and micromechanical pump in drug delivery system.

In recent years, it has been found that many other few‐layer metal elements and their oxides have unique optical properties,^[^ ^60^ ^]^ and the optical biosensors based on them will become an important basis for medical treatment and pollution prevention.

### 4.6 Optical Biosensors Based on 2D Degenerate Semiconductors

**Figure 23:**
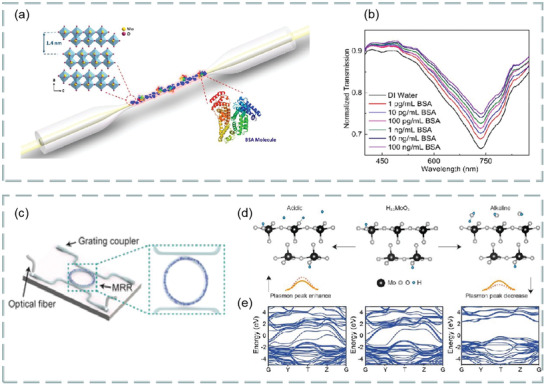
a) Structure diagram of optical biosensor. b) Transmission spectra at different concentrations of BSA. Reproduced with permission.^[^ ^174^ ^]^ Copyright 2018, American Chemical Society. c) Structure diagram of the micro‐ring resonator. d) Schematic diagram of optical sensing mechanism. e) Changes in electronic band structure of the synthesized H_0.3_MoO_3_; (left) Samples exposed to acidic environment with high H^+^ dopant concentration; (right) Sample exposed to extreme alkaline environment and all H^+^ dopants are completely extracted. Reproduced with permission.^[^ ^175^ ^]^ Copyright 2019, Wiley‐VCH.

Degenerate doped semiconductors are a new kind of plasma materials, and their plasma characteristics can be controlled by the concentration of dopants in the crystal structure.^[^ ^63^ ^]^ Compared with traditional plasma precious metals, their free carrier concentration is relatively small, and the disturbance of carrier density in doped semiconductors will affect the spectral position of plasma. Importantly, this unique characteristic is helpful to apply this plasma crystal to detect charge transfer in biochemical processes, and can develop charge‐sensitive biosensors. For example, Zhang et al. proposed a method to synthesize several heavily doped free electrons by H^+^ intercalation *α*‐MoO_3_ nanosheets, the resulting sub‐stoichiometric MoO_3‐_ *_x_* nanosheets provide strong plasma resonance with a wavelength of ≈735 nm.^[^ ^174^ ^]^ Study found that positively charged MoO_3‐_ *_x_* nanosheets show stable connection with polyanion functionalized microfibers and good affinity with negatively charged biomolecules. As shown in **Figure** **23**a, the surface of the microfiber is covered with a thin layer of heavy doping *α*‐MoO_3_. Among them, the strong evanescent field of microfiber effectively excites the surface plasma of MoO_3_ nanolayer. Then, bovine serum albumin (BSA) molecules are fixed on the surface of MoO_3_ nanosheet and interact with surface plasma, resulting in the change of transmission spectrum. In addition, to stabilize and fix MoO_3‐_ *_x_* nanoflakes, polyelectrolyte was used to functionalize the surface of optical fiber. As we know, BSA is a negatively charged protein. Thus, when applied to the detection of BSA, it can be effectively adsorbed on the surface of MoO_3‐_ *_x_* nanosheets through electrostatic interaction and van der Waals force. With the detection of increased BSA concentration, the detection limit of bovine serum albumin as low as 1 pg mL^−1^ was finally achieved, as shown in Figure 23b. This study confirmed the feasibility and prospect of the application of 2D MoO_3‐_ *_x_* plasma nanosheets in highly integrated equipment.

In addition, Ren et al. also proposed a silicon photonic ion sensor based on plasma material driven by ion dopant,^[^ ^175^ ^]^ as shown in Figure 23c. Clearly, this sensor consists of a microring resonator (MRR) and a 2D repackaged near‐infrared plasma molybdenum oxide layer (2D H*_x_* MoO_3_). In principle, when the 2D plasma layer interacts with ions from the environment, the strong change of refractive index leads to the shift of MRR resonance wavelength, and the change of plasma absorption leads to the modulation of MRR transmission power. Thus, the proof of concept through the pH sensing model is demonstrated. Meanwhile, the doping driving plasma characteristics of 2D H_0.3_MoO_3_ are affected by ion type and concentration, ion sensing of integrated sensor is achieved by exposure to different pH values, as shown in Figure 23d. When the solution is more acidic, the surrounding H^+^ concentration increases, the ions can diffuse and finally insert into the surface of 2D H_0.3_MoO_3_ at a smaller potential. Subsequently, the plasma absorption driven by H^+^ dopant is enhanced, resulting in additional transmission loss of MRR. In addition, injecting electrons while H^+^ doping makes the plasma compound more metallized, which will affect its dielectric properties and the refractive index. On the other hand, when the solution becomes more alkaline, OH^−^ ions dominate and tend to react with the H^+^ dopant of 2D H_0.3_MoO_3_ to form water molecules and release them from the structure. Figure 23e shows the change of electron band structure as a function of H^+^ doping concentration in H_0.3_MoO_3_. Clearly, the intensity of the plasma absorption peak is reduced due to the extraction of H^+^ from the main structure, so the MRR transmission is improved. Compared with conventional optical pH sensors, its sensitivity per unit area is improved by seven orders of magnitude in the range of 1 to 13.

High concentration doped MoS_2_ is also a 2D degenerate semiconductor material suitable for SPR technology. For example, Wang et al. embedded lithium into 2D MoS_2_ nanosheets by electrochemical method to realize plasma resonance in the visible and near‐ultraviolet wavelength range, and used the system to benchmark the biosensor with BSA.^[^ ^176^ ^]^

## 5 Application of 2D Material‐Based Optical Biosensors

In recent years, with the rapid development of optical biosensors based on 2D materials, they have been more and more applied in many fields, such as biological imaging, environmental pollution prevention/control, and biomedicine.^[^ ^177^, ^178^ ^]^ Next, we will discuss the applications of these sensors in detail.

### 5.1 Biological Imaging

**Figure 24:**
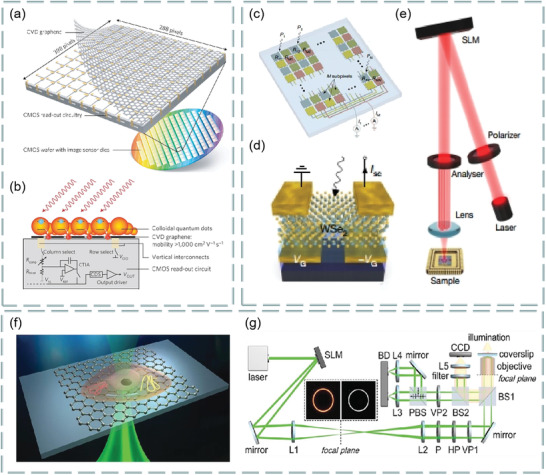
Biological imaging sensor based on 2D materials. a) CMOS–graphene quantum dot image sensor. b) Side view of graphene photoconductor and readout circuit. Reproduced with permission.^[^ ^179^ ^]^ Copyright 2017, Springer Nature. c) Artificial neural network photodiode array. d) Practical artificial neural network image device based on WSe_2_. e) Schematic diagram of optical setting. Reproduced with permission.^[^ ^181^ ^]^ Copyright 2020, Springer Nature. f) Schematic diagram of refractive index sensing of living cells on graphene surface. g) Schematic diagram of optical system structure. Reproduced with permission.^[^ ^182^ ^]^ Copyright 2018, The Royal Society of Chemistry.

Biological imaging based on optical sensors plays an important role in food safety, environmental monitoring and medical treatment, especially in various imaging systems. For example, Goossens et al. proposed a complementary metal‐oxide semiconductor (CMOS) integrated circuit and graphene monolithic integration as a high mobility phototransistor to prepare a high‐resolution, broadband image sensor and make it part of a broadband digital camera sensitive to ultraviolet, visible and infrared light (300–2000 nm).^[^ ^179^ ^]^ In the experiment, the image sensor based on 388 × 288 graphene quantum dot photodetector array shows integration potential, as shown in **Figure** **24**a. It can be seen that the process starts with transferring graphene to a CMOS chip containing the readout circuit of the image sensor. Figure 24b shows a side view of a graphene photoconductor and a readout circuit. Study found that graphene channels are sensitive to ultraviolet, visible, near‐infrared, and short‐wave infrared light through colloidal quantum dots. In principle, when the light is absorbed, the electron–hole pair will be generated due to the built‐in electric field, and the hole will be transferred to graphene and the electrons are retained in the colloidal quantum dots. Importantly, this high detection rate, the spectral sensitivity of 300–2000 nm and switching time of 0.1–1 ms verify the applicability of infrared imaging. It should be noted that in the package of the device, in addition to the photosensitive pixels, the imager also includes a row of blind pixels to subtract the dark signal, and the spectral range is determined by the material and size of the quantum dots. In the future, this kind of image sensor can be designed to operate in higher resolution and wider wavelength range, and may even have an overall size suitable for smart phones or smart watches, so it is expected to become a competitive image sensor with millions of pixel resolution and pixel spacing as low as 1 µm. Meanwhile, the future development of graphene transfer and packaging (e.g., based on hexagonal boron nitride^[^ ^39^ ^]^) will further improve the uniformity and performance of graphene‐based CMOS technology.

Using imaging to diagnose and detect cancer cells is a common medical method. Thus, the detection and identification of rare circulating tumor cells in patient's blood are of great significance for cancer diagnosis and monitoring. At present, bright field microscope images are generally used for data analysis. Study found that convolutional neural network can be used to label the cells detected in microscopic images of blood samples containing leukocytes and circulating tumor cell patients.^[^ ^180^ ^]^ Importantly, as a machine learning algorithm, artificial neural network has achieved great success in many fields. For example, Mennel et al. used WSe_2_ to study artificial neural network image sensing.^[^ ^181^ ^]^ Based on a reconfigurable 2D semiconductor photodiode array, this sensor can perceive and process optical images without delay. As shown in Figure 24c, it is composed of N active pixels arranged in a 2D array. In principle, each pixel is divided into M subpixels, which work under short‐circuit conditions and emit photocurrent under illumination:

(10) $$I_{\mathrm{mn}}=R_{\mathrm{mn}}E_{n}A=R_{\mathrm{mn}}P_{n}$$

where *R*_mn_ is the sub‐pixel light response rate; *E*_n_ and *P*_n_ are the local irradiance and optical power at N pixels respectively, and *A* is the detector area.

Using a schematic diagram based on WSe_2_, as shown in Figure 24d, it operates under short‐circuit conditions and provides a light response by supplying voltage to the bottom gate electrode of *V*_G_/*V*_G_. For the training and testing of the designed chip, the projection optical image is used, as shown in Figure 24e. Clearly, the laser is linearly polarized by a line grating polarizer and selected by a spatial light modulator (SLM). Notably, the reference light is filtered through the analyzer (intensity modulation) and the projected image is projected onto the photodiode array. In the experiment, a number of layers of WSe_2_ crystals with a thickness of about 4 nm are used to form a transverse p–n junction photodiode. The choice of WSe_2_ is due to its bipolar conduction behavior and excellent photoelectric properties. In addition, the technology is then used to produce the photodiode array as shown in Figure 24b, which has good uniformity, tunability and linearity from the 27 detectors. These devices are arranged in a 3 × 3 imaging array (*n* = 9), pixel size is about 17 µm^2^, with three detectors per pixel (*M* = 3).

Real‐time noninvasive living cell microscope is an important part of medical research. It is found that due to the different reflectivity of the two polarization states of graphene and 45° generalized cylindrical vector, the reflected cylindrical vector beam can be demodulated by laser to realize differential detection. Thus, subcellular refractive index imaging technology based on graphene biosensor systems came into being. Specifically, it is a label‐free and damage‐free refractive imaging technology based on vector beam, common light path focusing and differential detection, especially suitable for microscopic imaging detection of living cells. At present, it has become an effective tool in biomedical fields such as clinical application and pathological research,^[^ ^182^ ^]^ as shown in Figure 24f,g. Figure 24f is a schematic diagram of refractive index sensing of living cells on the surface of graphene, and Figure 24g is a schematic diagram of the structure of the optical system. Because the generated beam has good imaging spatial resolution and refractive index sensitivity, it has potential in the accuracy of detecting living cells. Specifically, the polarization beam splitter (PBS) divides the beam into horizontal and vertical linear polarization components, and then receives two beams of light respectively using a balance detector (BD) to record the absorption difference between S polarization and p polarization of graphene. In principle, because the scattering scanning near‐field optical microscope tip greatly enhances the local SPR mode, the electrons emitted by the tip are coupled to form asymmetric surface plasmon sub stripes in the vertical excitation configuration. Furthermore, dipole and higher‐order surface plasmon resonance modes can be observed by changing the width of graphene nanoribbons. These results provide a new idea for the study of the resonant behavior of new nanophotonic media other than graphene and are expected to be applied to biological imaging.

Photoacoustic imaging is a hybrid noninvasive biomedical imaging technology that irradiates nonionizing laser pulses onto biological tissues. Specifically, it generally generates ultrasonic signal (photoacoustic signals) by tissue absorbing light, and this photoacoustic signal carries the light absorption characteristic information of tissue, and then the light absorption image can be reconstructed by detecting it. Study found that MnO*_x_*/Ta_4_C_3_‐soybean phospholipid composite nanosheet has high photothermal conversion capability, and the photoacoustic signal has a linear relationship with the concentration of Ta in MnO*_x_*/Ta_4_C_3_‐soybean phospholipid, indicating that it has good photoacoustic imaging ability.^[^ ^183^ ^]^ Due to its low tissue attenuation coefficient, MXene‐based photoacoustic imaging is expected to break through the limitations of traditional optical imaging technology and become a promising imaging method, so as to provide guidance and evaluation for real‐time monitoring of biological structure and imaging of treatment process.

Optical coherence tomography is a sensing method widely used in medical imaging. In particular, it uses low coherence interferometry to generate 2D images of light scattering of internal tissue microstructure in the form of ultrasonic pulse‐echo imaging.^[^ ^15^, ^184^ ^]^ For example, Farid et al. used optical coherence tomography to study the biological pollution resistance and mechanism of graphene oxide surface coating, which showed that this membrane had good antibacterial activity against the proliferation of planktonic cells.^[^ ^185^ ^]^ It is believed that the combination of optical coherence tomography technology and graphene oxide can open up a new way to inhibit the initial growth of bacteria and subsequent biofilm formation.

### 5.2 Food Safety and Environmental Pollution Prevention/control

**Figure 25:**
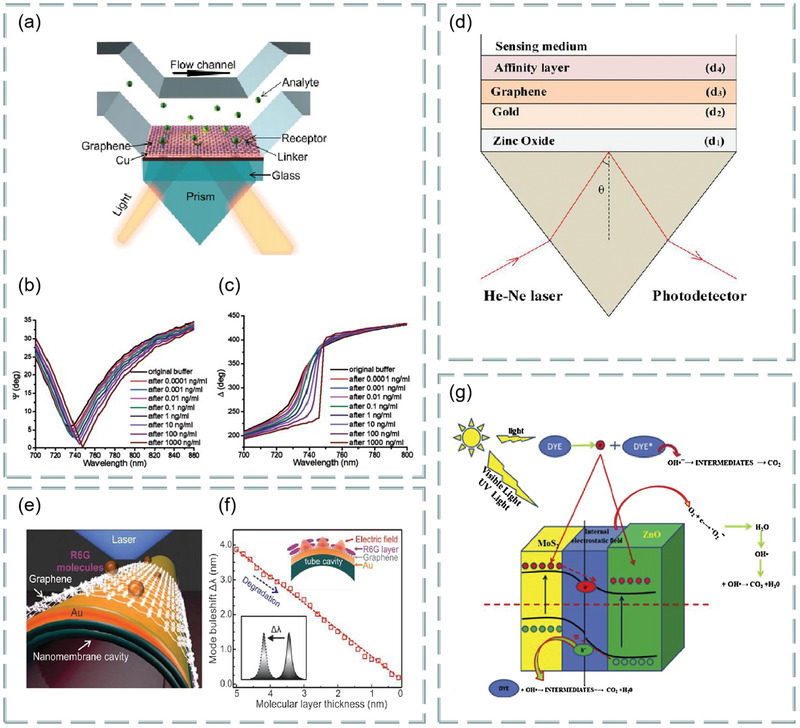
Food safety and environmental pollution prevention/control. a) Schematic diagram of optical biosensor based on SPR. b) SPR spectra of different concentrations of HT‐2 in phosphate‐buffered saline and the variation of amplitude *ψ* with the concentration of HT‐2. c) SPR spectra of different concentrations of HT‐2 in phosphate‐buffered saline and the variation of phase (Δ) with the concentration of HT‐2. Reproduced with permission.^[^ ^187^ ^]^ Copyright 2019, Springer Nature. d) Structure of SPR sensor for bacterial detection. Reproduced with permission.^[^ ^188^ ^]^ Copyright 2018, Elsevier. e) Schematic diagram of graphene activated photoplasma cavity based on coiled nanofilm. f) Mode shift of R6G molecular layer thickness with degradation. Reproduced with permission.^[^ ^189^ ^]^ Copyright 2019, American Chemical Society. g) Schematic illustration of photocatalytic mechanism of nanocomposites. Reproduced with permission.^[^ ^190^ ^]^ Copyright 2019, Elsevier.

Food safety is one of the key topics in the 21st century. Rapid detection of target bacteria and viruses, such as identification and quantification of *Escherichia coli* in water in complex food industry, is very important for providing safe food supply and preventing foodborne diseases.^[^ ^186^ ^]^ For example, Wu et al. used plasma biosensor technology to detect various molecular species in solution,^[^ ^187^ ^]^ as shown in **Figure** **25**a. In principle, when toxins bind to the surface within the polarization field of electric surface plasmon polaritons, they will change the local refractive index near the metal surface, thus changing the SPR characteristics directly detected by ellipsometry. Figure 25b,c illustrates the SPR spectra of phosphate‐buffered saline after different concentrations of HT‐2, that is, the variation of ellipsometry parameters (amplitude and phase) with concentration. This study shows that the passivation and functionalization of layered materials such as graphene can broaden the metal detection range of plasma biosensors, such as low molecular weight HT‐2 toxin, which paves the way for the realization of new high‐sensitivity biosensors and provides convenience for real‐time nursing detection.

Pseudomonas and its species in the product may cause serious food poisoning and serious infection (blood, lungs, skin, ears, and eyes) to hospitalized patients or patients with weakened immune system. Recently, SPR biosensors based on ZnO, gold, and graphene have been found to have good performance in the detection of *Pseudomonas*. For example, Kushwaha et al. used water as the sensitive medium of the sensor and applied ZnO, gold, graphene, and different affinity layers on the prism substrate,^[^ ^188^ ^]^ as shown in Figure 25d. Among them, the molecular recognition sites on graphene are tightly bound by the affinity layer and *Pseudomonas*; meanwhile, zinc oxide causes a large offset of the resonance angle, which significantly improves the sensitivity of the biosensor. Thus, it is expected to have important value in food safety.

Environmental pollution is one of the three major crises in the world, and the application of optical sensors in biological pollution prevention and control has also brought great opportunities to people. For example, Yin et al. proposed graphene‐activated photoplasma nanoscale cavities for photodegradation detection,^[^ ^189^ ^]^ as shown in Figure 25e,f. In principle, the photodegradation kinetics of organic dye molecules at the molecular level is monitored in real‐time by using graphene activated photoplasma chamber based on crimped nanomaterials. Specifically, the degradation process of Rhodamine 6G molecule on the surface of graphene activated sensor was monitored by laser local irradiation and optical resonance displacement measurement. Figure 25f shows the mode drift of R6G molecular layer thickness varying with degradation in graphene‐activated plasmas. Thus, this technology lays a foundation for the further study of resonance photocatalytic degradation mechanism and has potential application prospects in the prevention/control of environmental pollution.

Industrial pollution is one of the greatest threats in today's environment. To solve this problem, Krishnan et al. used MoS_2_/ZnO nanocomposites as a photocatalyst to degrade industrial pollutants such as methylene blue.^[^ ^190^ ^]^ Study found that the photocatalytic efficiency was determined by reducing the concentration of methylene blue under ultraviolet–visible light, as shown in Figure 25g. In principle, there are two ways of dye degradation: First, when the energy ray from the sun/light source irradiates the dye, the dye molecules are transformed into reactive dye molecules by transferring their electrons to the conduction bands of MoS_2_ and ZnO, and these molecules further enter the transition state of the electrostatic field formed at the heterojunction; the second is the formation of hybrid junction, which not only improves the separation of electron–hole pairs, but also enhances the photocatalytic and degradation process, especially the electrons in ZnO stimulated by ultraviolet light and MoS_2_ stimulated by visible light. Under the action of electrostatic field, the conduction electrons of MoS_2_ can easily migrate to ZnO. Note that this structure requires obvious separation of electrons and holes at the interface, so as to prolong the life of carriers, prevent the recombination of electron–hole pairs, and finally achieve the expected output of enhancing photocatalytic ability.

Water is the source of life, and now water pollution is becoming more and more serious. To deal with this, Tang et al. proposed an evanescent wave adaptive sensor based on target binding to promote fluorescence quenching by using FRET technology,^[^ ^191^ ^]^ which is used for online continuous and population‐specific detection of aminoglycoside antibiotics, in which graphene oxide is used as fluorescence quenching agent. In principle, the DNA aptamer labeled with fluorophore forms a multichain complex without aminoglycoside antibiotics, and the aminoglycoside antibiotics combine with aptamer to form aminoglycoside antibiotic aptamer complex. Furthermore, the fluorescence between fluorophore and aminoglycoside antibiotics further induces electron transfer and partially quenches the fluorescence of aminoglycoside antibiotic aptamer complex. Study found that the selective adsorption of aminoglycoside antibiotic aptamer complex on graphene oxide further inhibited the fluorescence of aminoglycoside antibiotic apt. This fluorescence quenching evanescent wave adaptive technology can be applied to the on‐site, continuous, and real‐time monitoring of pollutants in environmental water samples. In addition, the hexagonal photonic crystal fiber sensor based on SPR effect also has broad application prospects in the fields of water quality monitoring, biosensor, and food safety.^[^ ^192^ ^]^

### 5.3 Biomedical Applications

Because of its high resolution and sensitivity, optical biosensor has become the most promising detection and diagnosis method in biomedical applications. Next, we will discuss their progress in cancer detection and treatment, as well as the detection of biomacromolecules, micro‐organisms, and biological nerves.

**Figure 26:**
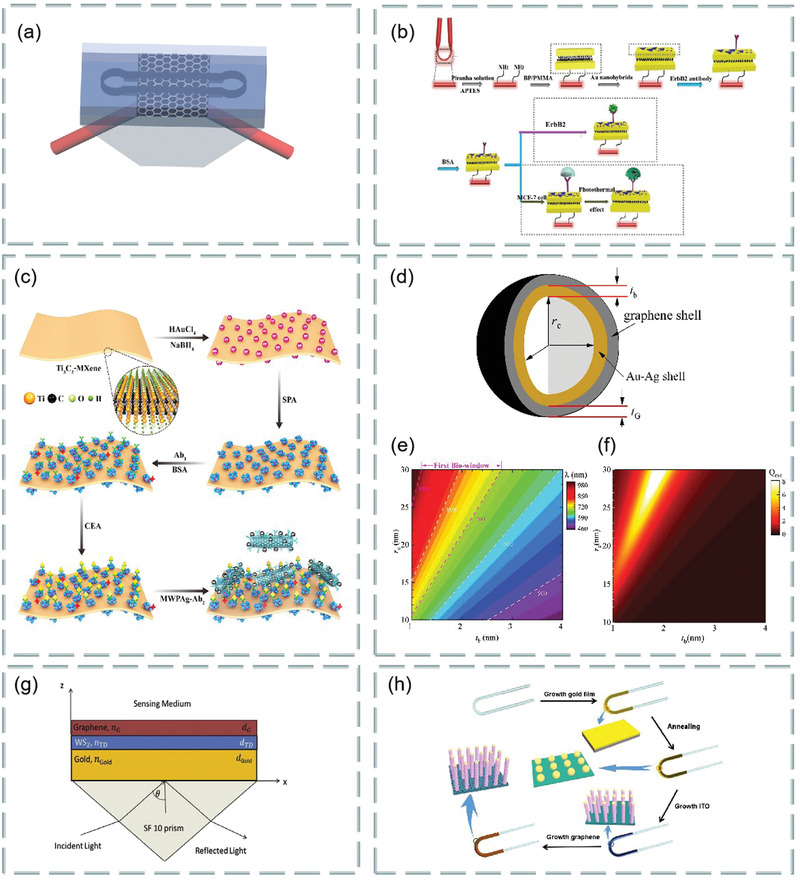
a) Reduced graphene oxide‐based optical refractive index sensor for cell detection. Reproduced with permission.^[^ ^50^ ^]^ Copyright 2014, American Chemical Society. b) Microfiber sensor with black phosphorus supported gold nano‐interface. Reproduced with permission.^[^ ^193^ ^]^ Copyright 2019, AAAS. c) Detection procedure of hypersensitive SPR biosensor for detection of carcinoembryonic antigen. Reproduced with permission.^[^ ^197^ ^]^ Copyright 2019, Elsevier. d) Graphene‐coated Au–Ag alloy hollow nanoshell structure. e) SPR peak wavelength of monolayer graphene‐coated Au–Ag alloy hollow nanoshells. f) Density diagram of extinction efficiency as a function of internal radius and Au–Ag shell thickness. Reproduced with permission.^[^ ^195^ ^]^ Copyright 2019, American Chemical Society. g) Surface plasmon resonance sensors for DNA hybridization with graphene WS_2_ coatings. Reproduced with permission.^[^ ^202^ ^]^ Copyright 2018, Elsevier. h) Process of synthesizing graphene/ITO nanorods metamaterial/U‐bend anneal sensor. Reproduced with permission.^[^ ^203^ ^]^ Copyright 2019, MPDI.

Cancer cells are cell variants and the source of cancer. Unlike normal cells, cancer cells have three characteristics: unlimited proliferation, transformation, and easy metastasis, so they can proliferate and destroy normal cells indefinitely. Thus, it is very important to sensitively detect and distinguish cancer cells from normal cells. In recent years, advanced biosensing technology based on graphene and its derivatives provides a new way for the detection and early detection of cancer cells.^[^ ^194^, ^195^ ^]^ For example, Xing et al. designed an optical refractive index sensor with high resolution, high sensitivity, and high dynamic range by using the polarization‐dependent absorption characteristics of reduced graphene oxide based on total internal reflection,^[^ ^50^ ^]^ as shown in **Figure** **26**a. In the experiment, at the single‐cell level, a small number of cancer cells in normal cells can be labeled, and living cells can be detected with high precision. In particular, the two cell lines can be detected and differentiated at the same time without separation. In addition, graphene and its derivatives can also be used as catalysts or supports for the most dangerous prostate cancer and show ultrahigh sensitivity.^[^ ^196^ ^]^

Nanointerface sensitized microfiber sensor can distinguish cancer cells from normal cells, which is suitable for early detection of cancer. However, the sensitivity of the traditional sensor is low, and it is expected to significantly improve the sensitivity of the sensor by modifying gold nanoparticles with black phosphorus,^[^ ^193^ ^]^ as shown in Figure 26b. Through the continuous modification of special solution, the researchers gave the surface charge of microfiber, and fixed the black phosphorus nanosheet coated with double‐layer polymethylmethacrylate on the surface of microfiber through electrostatic adsorption, so as to carry out cell photothermal therapy on cancer cells. This work opens up a possible way for the integration of cell diagnosis and treatment.

Besides graphene and black phosphorus, other 2D materials are also widely used in cancer detection. For example, Wu et al. used Ti_3_C_2_ MXene to improve sensitivity and proposed an ultrasensitive SPR biosensor for detecting carcinoembryonic antigen (CEA),^[^ ^197^ ^]^ as shown in Figure 26c. Specifically, the detection procedure of the biosensor is to dilute different concentrations of CEA with PBS and incubate in the mobile cell of immobilized CEA antibody (Ab1) sensing membrane for 30 min, and then inject PBS into the mobile cell to remove unbound CEA. After the resonance angle was stabilized, the multi‐walled carbon nanotube polydopamine silver nanoparticle composite was injected into the flow cell and maintained for 20 min, and the sensor response before and after PBS flushing was measured.

Besides the above‐mentioned cancer detection, optical sensors based on 2D materials also have great potential in cancer treatment. For example, it was found that MoS_2_–ZnO nanocomposites exhibit significant excitation wavelength‐dependent down‐conversion and up‐conversion photoluminescence, and can induce apoptosis and inhibit tumor growth by specifically activating caspase‐3.^[^ ^198^ ^]^ In recent years, it has been found that photothermal therapy is a minimally invasive technology, in which the incident light is absorbed by nanoparticles and its energy is transformed into local heat in tumor tissue, so as to selectively kill cancer cells and induce their apoptosis, or directly destroy cancer cells by rapid and severe death at local temperature. For example, Farokhnezhad et al. Studied the optical and photothermal properties of graphene coated gold silver alloy hollow nanoshell and found that it is suitable for a class of excellent photothermal treatment nanomaterials,^[^ ^195^ ^]^ as shown in Figure 26d. In the experiment, metal nanoparticles have great photothermal conversion efficiency and excellent optical properties, which are suitable for photothermal therapy. Thus, if nanoparticles in tumor tissue are irradiated by laser, their plasmons will be excited, so as to absorb light energy and finally dissipate into heat. Specifically, the researchers adjusted the SPR peak of gold and silver alloy hollow nanoshell (Au‐Ag‐HNSs)‐coated graphene in the biological window by changing the thickness of Au–Ag and graphene layers and the composition of Au–Ag shell alloy. Furthermore, nanoparticles with SPR peaks are used in the biological window to reduce the duration and side effects of cancer treatment, so as to ensure the effectiveness of photothermal therapy. The SPR peak wavelength density and extinction efficiency of single‐layer graphene‐coated gold and silver alloy hollow nanoshells are shown in Figure 25e,f.

MXene is also suitable for photothermal therapy tumor therapy. For example, Han et al. used Ti‐MXene quantum dots for near‐infrared thermotherapy.^[^ ^199^ ^]^ Study found that the surface temperature of tumor tissue increased to 60 °C within 5 min after laser irradiation, which was sufficient to abate the tumor. Notably, in animals treated with Ti‐MXene quantum dots and near‐infrared radiation, the tumor was completely eliminated within 14 d without recurrence, which proves that 2D material has a good effect in cancer treatment. In addition, in drug delivery, 2D nanomaterials, like many other types of nanoparticles, can effectively attack cancer cells by controlling drug release and enhancing cell absorption of payload, and MXene is a good drug delivery medium.^[^ ^200^ ^]^

Biological macromolecule is one of the criteria for measuring a person's health, so its detection is a hot‐spot in contemporary medical research. Studies have found that gene‐testing can be used to diagnose diseases and predict disease risks. For example, DNA sequencing can determine the direction and structure of recombinant DNA, so it is of great significance for the diagnosis and treatment of genetic diseases and the development and delivery of drugs. Recently, Min et al. used graphene nanoribbons functionalized fluid nanochannels to analyze the changes of base conductivity to complete DNA sequencing.^[^ ^201^ ^]^ In the experiment, this sequencing method greatly shortens the sequencing time, can analyze 3 billion bases in one hour, and the efficiency is greatly improved.

In addition, Rahman et al. proposed a graphene‐WS_2_‐coated SPR sensor for DNA hybridization based on prism (SF10 glass),^[^ ^202^ ^]^ as shown in Figure 26g. In the experiment, the addition of graphene layer increases the sensitivity of the sensor, but reduces other performance parameters. In order to improve all performance parameters, WS_2_ was added between metal layer and graphene layer, and its sensitivity, accuracy and quality were improved. Interestingly, Wang et al. proposed a U‐based curved SPR sensor based on graphene/ITO nanorod metamaterial/U bending annealing for detecting target DNA,^[^ ^203^ ^]^ as shown in Figure 26h. This sensor has the primitive ITO nanocolumn array structure and coated graphene, so that it can produce significant bulk plasmon resonance effect. Importantly, its discontinuous structure not only produces a larger surface area for target DNA molecules, but also for more biological molecules, so as to give full play to biological characteristics and have a good prospect of medical application.

Glucose is the energy source and metabolic intermediate product of living cells. Excessive glucose can increase insulin concentration, leading to obesity and diabetes, and too little glucose can lead to hypoglycemia and even affect the brain. Recently, Liu et al. used g‐C_3_N_4_ and TiO_2_ nanosheet composites as scaffolds to construct photoelectrochemical enzyme biosensors for glucose detection.^[^ ^204^ ^]^ In the experiment, the weak visible‐light excitation of TiO_2_ was improved and the photocurrent charge recombination on g‐C_3_N_4_ was delayed, which enhanced the response of the photoelectrochemical biosensor. In addition, Yang et al. studied TiO_2_ nanotubes modified by polydopamine and graphene quantum dots and constructed an ultra‐sensitive photoelectrochemical double electron receptor biosensor.^[^ ^145^ ^]^ These two methods have been successfully applied to the development of photoelectrochemical glucose biosensor.

The balance between catalase and ascorbic acid is very important to the human body. Majumder et al. found that the circular gold nanosheets coated with multifunctional reduced graphene oxide greatly enhanced the photoluminescence emission intensity caused by the common wobble of SPR and the fluorescence resonance energy transfer effect through the enhancement of Raman spectrum intensity.^[^ ^205^ ^]^ Thus, under visible light irradiation, photocurrent flows through the sample to show the photocatalytic decomposition of water, indicating that it has great potential in the detection of nonenzymatic H_2_O_2_ and ascorbic acid.

**Figure 27:**
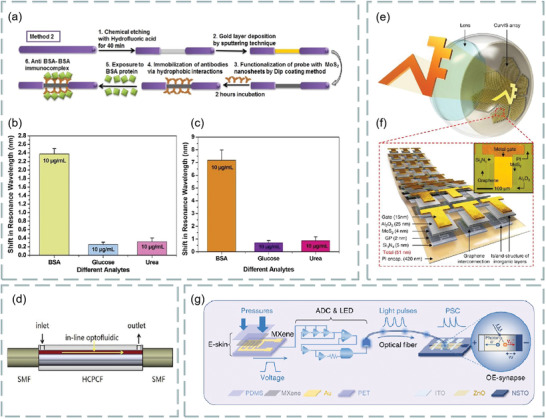
a) MoS_2_‐modified optical fiber SPR biosensor. b,c) Comparison of different compound specificity of optical fiber SPR biosensor with and without MoS_2_ coating in PBS solution. Reproduced with permission.^[^ ^7^ ^]^ Copyright 2019, Springer Nature. d) Photonic crystal fiber sensor based on anti resonant reflection waveguide. Reproduced with permission.^[^ ^207^ ^]^ Copyright 2017, Optical Society of America. e) Schematic diagram of high density curved image sensor array. f) Design of high density bending image sensor array. Reproduced with permission.^[^ ^210^ ^]^ Copyright 2017, Springer Nature. g) Design of mechanical sensor system in somatosensory system. Reproduced with permission.^[^ ^211^ ^]^ Copyright 2020, Springer Nature.

Bovine serum albumin (BSA) is widely used in biochemical research, genetic engineering, and medical research. Study found that the functionalization of optical fiber SPR probe and antibody can be used for label‐free detection of BSA protein. For example, Kaushik et al. proposed a MoS_2_‐modified optical fiber SPR biosensor,^[^ ^7^ ^]^ as shown in **Figure** **27**a. Specifically, gold‐plated optical fibers were modified with MoS_2_ nanosheets, and then biofunctionalized with anti BSA antibodies. This technology not only amplifies SPR signal through the synergistic action of MoS_2_ and metal film, but also adheres directly to representative antibodies through hydrophobic action without chemical reaction. Thus, it is expected to be used to develop optical devices for monitoring various biomedical and environmental parameters. Figure 27b,c shows the optical fiber SPR biosensor with or without MoS_2_ coating. Furthermore, by comparing the specificity of different compounds in PBS solution, it is found that the sensitivity of MoS_2_ sensor has been greatly improved. In addition, the sensitivity of bimetallic (Al/Au) SPR sensor is much higher than that of a traditional SPR sensor. By studying the optimal coupling response of different prisms (BK7 prism), the sensor based on the optimal thickness of black phosphorus, graphene, and WS_2_ can be effectively used for biomolecular interaction detection and medical diagnosis.

Microbial detection is also a hot topic in the field of biomedicine. For example, Rodrigo et al. studied different Escherichia coli strains by electrophoretic deposition at the gold‐based SPR interface with reducing graphene oxide film.^[^ ^112^ ^]^ This is because the integration of different *Escherichia coli* strains with affinity targets can be easily realized due to the noncovalent interaction between graphene oxide matrix and various organic ligands. In particular, it is not only *Escherichia coli*, but also applicable to any other bacteria.

For virus detection, waveguide‐coupled 2D photonic crystal is used as a sensor to detect virus‐sized particles in fluid flow.^[^ ^206^ ^]^ For example, to detect unlabeled biological molecules such as bacteria and DNA, Gao et al. proposed an anti‐resonance reflection waveguide coated with multilayer graphene,^[^ ^207^ ^]^ as shown in Figure 27d. The effective refractive index of graphene layer is adjusted by the resonance condition of Fabry‐Perot resonator and visible laser beam heating. Meanwhile, photonic crystal fiber (PCF) and hole in photonic crystal fibers provide natural online optical jets. This scheme of detecting refractive index and liquid velocity by using wavelength shift and visibility in transmission spectrum provides a simple and real‐time method for studying biomolecular interaction dynamics.

Inhibiting the growth of certain microorganisms is essential. To this end, the medical community has used different antibacterial agents. However, the overuse of these bactericides will lead to the gradual drug resistance of bacteria, thus neutralizing the normal function of antibacterial molecules. Recently, it has been found that 2D nanomaterials (transition metal carbides, carbides, and nitrides), such as MXene, are suitable as antibacterial agents to inhibit the growth of bacteria and fungi because of their increased membrane permeability, cell membrane rupture, reduced metabolic activity and DNA damage.^[^ ^208^ ^]^

Biological nerve detection is an indispensable part of biomedicine. Study found that, by integrating electrical, optical and chemical stimulation modes into a large number of nerve probes, we can study the important potential mechanisms of brain diseases. Thus, Son et al. proposed the optical modulation of nerve signals in transgenic mice through compact 2D MEMS neural array,^[^ ^209^ ^]^ which confirmed the function of 2D photodiode array and its potential as the next generation optogenetics application. Although these have made significant progress in the perception and regulation of neural activity methods and devices, it is still necessary to further improve the temporal and spatial resolution, cell type selectivity, and long‐term stability of neural interface. In addition, implantable optoelectronic devices for optical sensing and retinal stimulation also provide new opportunities for the next generation of biological optic nerve detection. For example, Choi et al. proposed a high‐density hemispherical bending image sensor array, which is designed with few‐layer MoS_2_–graphene heterostructure and strain release device,^[^ ^210^ ^]^ as shown in Figure 27e. It can detect optical signals and electrically stimulate the optic nerve with minimal mechanical side effects on the retina. Figure 27f shows a schematic diagram of the device design, and the illustration shows an optical microscope image of a single phototransistor. Clearly, the high‐density bending image sensor array (CurvIS) is a promising soft retinal implantation imaging element, and it is also a soft implantation optical device inspired by human eyes. In principle, near‐infrared light stimulation can increase the activities of visual cone cells, ganglion cells, and cortical neurons, so as to realize light‐driven behavior. Due to the sensitivity of near‐infrared light to the blind retina, it is expected to provide the possibility for the blind to see light again in the future.^[^ ^87^ ^]^

In the somatosensory system, the integration and cooperation of mechanical receptors, neurons, and synapses enable human beings to effectively perceive and process tactile information. For example, Tan et al. proposed a photoelectric stimulation afferent nerve based on MXene material, which has the abilities of perception, neural coding, perceptual learning, and memory,^[^ ^211^ ^]^ as shown in Figure 27g. Specifically, by coupling the light‐emitting diode (LED) to the ring oscillator and the edge detector, the detected information is converted into light stimulation and coding, and then integrated through the optical pulse coding integrated by the photoelectric synapse. The system can not only detect the pressure information, but also recognize Morse code, Braille and object motion.

**Figure 28:**
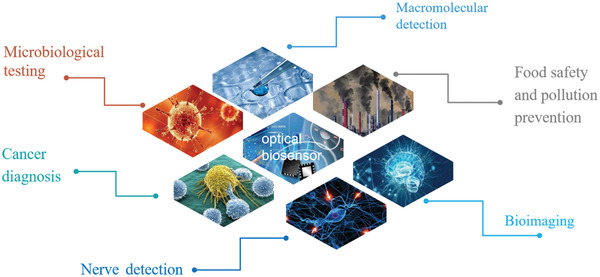
Application of optical biosensor based on 2D materials.

These different kinds of optical biosensors will become an important cornerstone in the field of sensors in the future, and their development trend is noninvasive, integrated, and intelligent. **Figure** **28** summarizes the applications of various optical sensors based on 2D materials in different biological fields. Because 2D materials are suitable for high‐level surface interaction with biological macromolecules, optical sensors based on them will be favored by more and more researchers, and have broad application prospects in medicine and drug delivery systems.

## 6 Summary and Prospect

In terms of devices, we review the latest progress of various optical biosensors based on 2D materials. With the emergence and wide application of graphene and other types of 2D materials, more and more researchers began to explore their properties and devices related to optical biosensors.^[^ ^212^ ^]^ In addition, there are many sensing methods for optical biosensors. Besides the technologies mentioned above, many other technologies can also be applied to biosensors.^[^ ^213^ ^]^ It can be expected that optical sensors based on 2D materials have a very broad development prospect in the field of biology.

In terms of application, we mainly review the latest application progress of optical biosensors based on 2D materials in biological imaging, environmental pollution prevention/control and biomedicine. Studies have shown that many types of 2D materials usually show strong infrared light absorption ability, which makes them useful candidates for cancer photothermal therapy.^[^ ^73^ ^]^ For example, in the detection of biological macromolecules, FRET or SPR methods have been used to detect DNA, RNA, protein, and other substances, especially in genetics and drug therapy. Because most 2D materials have biocompatibility and biodegradability, their reliability and safety in multiple detection, accurate detection, and safety monitoring in biomedicine are guaranteed. It is worth mentioning that in the next few decades, the implantable photoelectric technology driven by new technology will also develop rapidly. In principle, it is a new injection photoelectric biosensor in biomedical electronics. It is mainly stimulated by light, and optical fiber plays a leading role in deep tissue stimulation. Specifically, reduce invasiveness by creating devices with nontraditional design and functions, accurately and safely locate human deep organs, and create new biosensor technologies.^[^ ^214^ ^]^ With the in‐depth development of 2D materials, their combination with implantable optoelectronic technology will become a new research direction.

**Figure 29:**
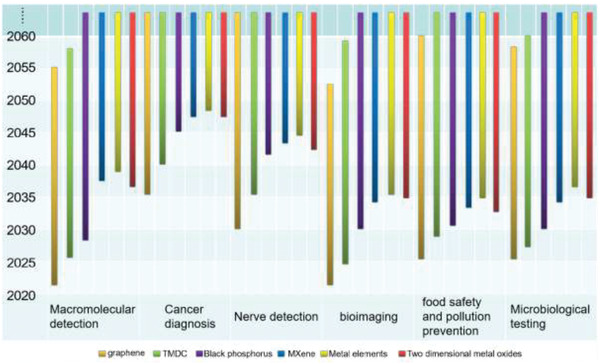
The roadmap of 2D materials based optical biosensors.

Nowadays, optical biosensors are developing in a diversified direction. Meanwhile, in the past few years, 2D materials other than graphene have developed rapidly. These materials provide new opportunities and challenges for biomedical and environmental monitoring applications. Although there are many researches on sensor devices based on 2D materials, most of them are limited to the laboratory, and the practical application requires a lot of time accumulation, especially in its reliability and reproducibility. **Figure** **29** summarizes the development prospects of 2D materials in different application directions. It can be expected that in the next 20–50 years, they will have a strong device research foundation and play an important role in practical applications.^[^ ^48^, ^70^ ^]^ More importantly, in the future, the 2D material optical sensor based on SPR, FRET, mid‐infrared and even terahertz and spin‐electron methods will become a new sensing platform and contribute to medical treatment, drug delivery, pollution prevention/control, and safety.

## Conflict of Interest

The authors declare no conflict of interest.
